# Mapping Alterations Induced by Long-Term Axenic Cultivation of *Leishmania amazonensis* Promastigotes With a Multiplatform Metabolomic Fingerprint Approach

**DOI:** 10.3389/fcimb.2019.00403

**Published:** 2019-12-04

**Authors:** Frederico Crepaldi, Juliano Simões de Toledo, Anderson Oliveira do Carmo, Leopoldo Ferreira Marques Machado, Daniela Diniz Viana de Brito, Angela Vieira Serufo, Ana Paula Martins Almeida, Leandro Gonzaga de Oliveira, Tiago Queiroga Nery Ricotta, Douglas de Souza Moreira, Silvane Maria Fonseca Murta, Ariane Barros Diniz, Gustavo Batista Menezes, Ángeles López-Gonzálvez, Coral Barbas, Ana Paula Fernandes

**Affiliations:** ^1^Clinical and Toxicological Analysis Department, School of Pharmacy, Federal University of Minas Gerais, Belo Horizonte, Brazil; ^2^Centro de Metabolómica y Bioanálisis, Unidad Metabolómica, Interacciones y Bioanálisis (UMIB), Universidad CEU San Pablo, Boadilla del Monte, Spain; ^3^Laboratory of Biotechnology and Molecular Markers, General Biology Department, Institute of Biological Science, Federal University of Minas Gerais, Belo Horizonte, Brazil; ^4^Manchester Institute of Biotechnology, The University of Manchester, Manchester, United Kingdom; ^5^René Rachou Research Center, Oswaldo Cruz Foundation, Belo Horizonte, Brazil; ^6^Morphology Department, Institute of Biological Science, Federal University of Minas Gerais, Belo Horizonte, Brazil

**Keywords:** *L. amazonensis*, attenuation, metacyclogenesis, inflammation, multiplatform metabolomic fingerprint, metabolic pathways

## Abstract

Leishmaniases are widespread neglected diseases with an incidence of 1.6 million new cases and 40 thousand deaths per year. *Leishmania* parasites may show distinct, species-specific patterns of virulence that lead to different clinical manifestations. It is well known that successive *in vitro* passages (SIVP) lead to the attenuation of virulence, but neither the metabolism nor the pathways involved in these processes are well understood. Herein, promastigotes of a virulent *L. amazonensis* strain recently isolated from mice was compared to SIVP derived and attenuated promastigotes, submitted to 10, 40, and 60 axenic passages and named R10, R40, and R60, respectively. *In vitro* assays and *in vivo* tests were performed to characterize and confirmed the attenuation profiles. A metabolomic fingerprint comparison of R0, R10, and R60 was performed by means of capillary electrophoresis, liquid and gas chromatography coupled to mass spectrometry. To validate the metabolomic data, qPCR for selected loci, flow cytometry to measure aPS exposure, sensitivity to antimony tartrate and ROS production assays were conducted. The 65 identified metabolites were clustered in biochemical categories and mapped in eight metabolic pathways: ABC transporters; fatty acid biosynthesis; glycine, serine and threonine metabolism; β-alanine metabolism; glutathione metabolism; oxidative phosphorylation; glycerophospholipid metabolism and lysine degradation. The obtained metabolomic data correlated with previous proteomic findings of the SVIP parasites and the gene expression of 13 selected targets. Late SIVP cultures were more sensitive to Sb^III^ produced more ROS and exposed less phosphatidylserine in their surface. The correspondent pathways were connected to build a biochemical map of the most significant alterations involved with the process of attenuation of *L. amazonensis*. Overall, the reported data pointed out to a very dynamic and continuous metabolic reprogramming process, accompanied by changes in energetic, lipid and redox metabolisms, membrane remodeling and reshaping of parasite-host cells interactions, causing impacts in chemotaxis, host inflammatory responses and infectivity at the early stages of infection.

## Introduction

Leishmaniasis is a group of neglected diseases, endemic in 98 countries, which are caused by different species of the genus *Leishmania*. Collectively, the incidence of leishmaniasis reaches 1.6 million new cases and ~40,000 deaths per year (WHO, 2010; Alvar et al., 2012; Courtenay et al., 2017). Among New World *Leishmania* species, *Leishmania amazonensis* has significant medical importance, being associated to all forms of leishmaniasis, including diffuse cutaneous leishmaniasis and visceral leishmaniasis, the former being a severe tegumentary form of the disease, while the latter is fatal if left untreated (Barral et al., 1991; Goto and Lindoso, 2010). *L. amazonensis* has evolved sophisticated mechanisms that allow resistance to extreme stress conditions, including oxidative burst and increased temperature in the host, as well as ways to subvert the host's immune system (McMahon-Pratt and Alexander, 2004; Soong et al., 2012; Carlsen et al., 2013).

To effectively achieve multiplication and transmission from one host to the other, *Leishmania* spp. undergoes several molecular, biochemical and morphological changes during its life cycle. The differentiation process undergone in the mammalian host involves the transformation of *Leishmania* from metacyclic promastigotes (introduced in the host's skin by the bite of a flebotominae sand fly) to amastigote forms (inside the host's macrophages). The differentiation process is more dynamic in the insect's gut and comprises several intermediate promastigote forms, including procyclic and metacyclic promastigotes. Metacyclogenesis is required for the detachment of promastigotes from the insect's gut and thus, effective transmission and infection of mammalian hosts (Bates and Rogers, 2004). While components on the insect's gut exert a selective pressure for maintenance of virulence factors (Sacks et al., 1985; da Silva and Sacks, 1987; McConville et al., 1992), successive *in vitro* passages (SIVP) are known to induce attenuation of virulence phenotype of *Leishmania* spp. (Moreira et al., 2012; Magalhães et al., 2014) and, accordingly, this procedure has been used to produce attenuated *Leishmania* for immunization (Saljoughian et al., 2014). Among several phenotypes that have been described, long-term axenic cultivation alters metacyclogenesis, amastigogenesis, calcium metabolism, and protease expression (da Silva and Sacks, 1987; Lu et al., 1997; Cysne-Finkelstein et al., 1998; Rebello et al., 2010; Moreira et al., 2012; Gomes et al., 2017). In agreement, a previous comparative proteomic analysis has revealed decreased virulence and several concomitant alterations in protein expression profile of *L. amazonensis* promastigotes generated through long-term SIVP (Magalhães et al., 2014). Recovery of virulence after *in vivo* passages has been also documented (Espiau et al., 2017). Moreover, it is possible to generate a diversity of sub-clones with reverting phenotypes from parasite clones with a given phenotype, disclosing the ability of continuous maintenance of parasite diversity for each *L. amazonensis* population (Espiau et al., 2017).

The -omics technologies have been widely applied in *Leishmania* studies to characterize parasite biology, disease phenotypes determinants and drug action mechanisms, as well as tools to discover new antigens (Paape et al., 2010; Coelho et al., 2012; Kaur and Rajput, 2014; Magalhães et al., 2014; McCall and McKerrow, 2014; Pawar et al., 2014). Despite previous–omics approaches, the mechanisms and the metabolic pathways related to SIVP and loss of virulence in *Leishmania* are still scantily described. Untargeted fingerprinting metabolomics has emerged as a powerful tool to unveil concomitant and global metabolic alterations occurring in a given biological condition, phenotype or in response to different stimuli (Lei et al., 2011). Fingerprinting studies usually generate a huge amount of data, requiring powerful bioinformatics analysis and additional metabolic and biological characterizations to be validated and integrated into pathways and with other–omics data to complement the information gathered from the post-genomic toolbox (Canuto et al., 2014; Villaveces et al., 2015). Metabolomics may have special relevance for *Leishmania*, due to the extremely high post-transcriptional regulation of gene expression (Canuto et al., 2014; Clayton, 2019). However, a single analytical system may be not sufficient to identify the broad range of metabolites potentially associated with such complex biological process (Canuto et al., 2014). Therefore, in this study we have applied a multi-analytical platform approach, encompassing liquid and gas chromatography and capilary eletrophoresis all coupled to mass spectometry (LC-MS, GC-MS and CE-MS, respectively) to identify as broadly as possible the highly diversified biochemical classes of metabolites associated with virulence attenuation in previously described SIVP parasites of *L. amazonensis* promastigotes (Magalhães et al., 2014). In parallel, additional assays were performed for biological characterization and validation of parasite infectivity, metacyclogenesis, phosphatidylserine exposure and sensitivity to antimonial drugs. This untargeted metabolomics approach unveiled and linked several metabolites and biochemical pathways that are, in a concerted and dynamic way, associated to the adaptation of promastigotes to long-term axenic cultivation and attenuation.

## Materials and Methods

### Ethics Statement

Experiments were performed in compliance with the National Guidelines of the Institutional Animal Care and Use Committee for the Ethical Handling of Research Animals (CEUA) from the Federal University of Minas Gerais (UFMG) (Law number 11.794, 2008), approved under protocol CETEA number 240/2016.

### Mice, Cells, and Parasites

Female BALB/c mice (8 weeks old) were obtained from the breeding facilities of the Department of Biochemistry and Immunology, Institute of Biological Sciences, Federal University of Minas Gerais, and were maintained under specific pathogen-free conditions.

The L929 cell line was kindly supplied by Dr. Miriam Testassicca (Federal University of Ouro Preto, Brazil). The cells were maintained at 37°C with 5% CO_2_ in DEMEM medium (GibcoBRL, Life Technologies) supplemented with L-glutamine 2 mM, HEPES 25 mM, Penicillin/100 IU/mL streptomycin and 100 mg/mL and 10% heat-inactivated fetal bovine serum (FBS).

The *L. amazonensis* strain (IFLA/BR/1967/PH-8) was originally provided by Dr. Maria Norma Mello, from the parasite collection of the Department of Parasitology, Federal University of Minas Gerais. All the SIVP cultures derived from this strain were provided by Dr. Eduardo Antonio Ferraz Coelho (Colégio Técnico, Federal University of Minas Gerais). These cultures were obtained as described by Magalhães et al. (2014). An axenic cell culture of the PH-8 strain, named R0, was recovered from the hind footpads of BALB/c mice after 8 weeks of infection and was used as the reference for a virulent profile. The other three derivate SIVP cultures, R10, R40, and R60 were obtained after 10, 40 and 60 successive *in vitro* passages (SIVP) from the R0 axenic culture. Parasites were grown at 26°C in M199 medium (GibcoBRL) at pH 7.4, supplemented with 10% heat-inactivated FBS, 20 mM L-glutamine, 100 UI/mL penicillin, 100 mg/mL streptomycin, adenine 10 mM, biotin 0.1% and hemine 0.25%.

### *In vitro* Infection

Bone marrow cell suspensions were obtained by washing the femurs of BALB/c mice with sterile 0.9% saline. Suspensions with 2 × 10^5^ parasites/mL were platted in Petri dishes in Complete Tissue Culture Medium (CTCM) [*Dulbecco's Modified Eagle's medium–*DMEM, supplemented with 10% inactivated BFS, L-glutamine 2 mM, penicilin G 100 UI/mL, 50 μM of β-mercaptoethanol (*Pharmacia Biotech, Uppsala*, Swiss) and HEPES 25 mM, pH 7.2]. L-929 cell conditioned medium (LCCM) was added (30%) at days 0 and 4 to induce cellular differentiation. At the 7th day of cultivation, promastigotes were incubated with BMMΦ at a 5:1 ratio. After 2 h, the infection was arrested and non-internalized promastigotes were removed with PBS (137 mM NaCl; 8 mM Na_2_HPO_4_; 2.7 mM KCl; 1.5 mM KH_2_PO_4_; pH 7.0). Infected cells were resuspended in CTCM at 8 × 10^5^ cells/mL and plated in *Lab-tek* chamber slides (NUNC, Thermo Fisher Scientific). Cells were stained using Panotic (Laborclin, Pinhais, PR, Brazil) and infection rates were determined at 0, 3, 12, 24, and 48 h after infection. A microphotographic system with 400–1000X magnification on an optical microscope (Olympus CH30 Binocular Microscope) was used to observe and photograph the cells. These assays were repeated three times to determine the reproducibility of experimental data. The number of infected cells was calculated by counting at least 300 cells per well (Boltz-Nitulescu et al., 1987). Differences among groups were determined by One-way ANOVA, and *p* < 0.05 were considered as significant.

### *In vivo* Infection

For *in vivo* infection, R0 and R60 promastigotes were collected at the 3rd day of the stationary phase and washed twice with cold PBS. The promastigotes were resuspended at 4 × 10^7^ cells/mL and 25 μL of this suspension was inoculated in the right hind footpad of female BALB/c mice (*n* = 10 per group). The footpads of each group were measured weekly to follow development of the lesion. Lesion size measurements (mm) were considered as the size of infected paw minus the non-infected one (left hind). At the 2nd week post-infection, the mice were euthanized to collect spleen cells for cultivation and supernatant for ELISA.

### Determination of Parasite Loads by Limiting Dilution Assay

Parasite burden in BALB/c mice hind footpads was determined by limiting dilution as previously described (Lima et al., 1997). Briefly, the footpads were homogenized in a douncer with M199 medium. Tissue debris was removed by centrifugation at 50 g at 4°C for 1 min. The supernatant was collected and centrifuged at 1540 g at 4°C for 10 min. The obtained pellet was resuspended in 500 μL of M199 medium and submitted to 1:10 serial dilution (in duplicate) in 96-well microplates at a final volume of 200 μL. Microplates were incubated at 26°C for 15 days and the presence of parasites was evaluated weekly. The final titer was considered as the last dilution in which at least one parasite could be identified in the well. Results were expressed as the negative logarithm of the number of parasites.

### Soluble *Leishmania* Antigen Preparation

*L. amazonensis* from early stationary phase cultures were collected and washed twice in sterile PBS. The obtained pellets were submitted to seven cycles of freeze-thaw in liquid nitrogen (thawing at 37°C). Preparations were observed on an optical microscope for the presence of intact parasites (de Souza et al., 2010). Protein content was determined by Bradford (1976) and adjusted to 1 mg/mL. Antigen suspension was stored in aliquots, at −80°C.

### Separation of *L. amazonensis* Promastigote Metacyclic Forms at Late Log (4th day) and Stationary (7th day) Phases From Axenic Cultures

#### Ficoll Gradient Separation Assay

A Ficoll gradient separation assay was performed using R0 and R60 promastigotes at the 4th and 7th days of cultivation. The cultures were separated in three sterile 15 mL conical tubes for each cell culture analyzed and the tubes were centrifuged at 50 g for 5 min at 4°C to remove dead parasites. Supernatants were transferred to new conical tubes and centrifuged at 1500 g at 4°C for 10 min. The obtained pellets were washed twice with 10 mL of cold PBS (4–8°C). After the second centrifugation, each pellet was suspended in Dulbecco's Modified Eagle's medium (DMEM) supplemented with 1% glucose (Sigma-Aldrich Inc.). 2.0 mL of suspensions with 1 × 10^7^ parasites/mL were distributed in three 15 mL conical tubes. With the assistance of a Pasteur pipette, 2.0 mL of Ficoll 10% (Ficoll Type 400, Sigma-Aldrich Inc.) was added at the bottom of the tube. This solution was centrifuged at 1300 g at 25°C for 15 min, using acceleration 1 and deceleration 0 (Spath and Beverley, 2001). Procyclic and metacyclic promastigotes were used to determine the gates for FACS assays.

#### Determination of Metacyclic Forms Population by Flow Cytometry

The *L. amazonensis* R0 and R60 promastigotes at the 4th and 7th days of growth were washed twice with PBS and fixed in 300 μL of 1% formalin. The populations of procyclic and metacyclic promastigotes were then analyzed by flow cytometry (BD LSRFortessa™ cell analyze) (10 thousand events) and identified with the Flowjo software, version 10.1, using the gates previously established with the parasites separated by Ficoll gradient (Supplemental Figure 2). Metacyclic forms were considered to have lower granularity and smaller size (SSC × FSC), when compared to procyclic promastigotes, as previously described (da Silva et al., 2015). Statistical analyses were performed with Two-way ANOVA with Bonferroni post-test.

#### *In vitro* Infection of Bone Marrow-Derived Macrophages (BMMΦ) and L929 Strain Cell Culture

Murine cell strain L929 producing macrophage colony-stimulating factor (M-CSF) was cultivated in CTCM and HEPES [25 mM, pH 7.2] at an initial concentration of 2 × 10^5^ cells/mL. Cells were incubated at 37°C with 5% of CO_2_. On the 3rd day of growth, the supernatant was collected, centrifuged (1540 g, 4°C, 10 min), sterilized by filtration and then stored at −80°C, until further use.

#### Preparation of BMMΦ

BMMΦ were obtained using the protocol described by Boltz-Nitulescu et al. (1987). BALB/c mice were euthanized by cervical dislocation. Their thighbone and shinbone were surgically dissected, submerged in 70°GL alcohol for 2 min and washed with PBS supplemented with 5% inactivated FBS. The epiphyses were removed and the diaphysis were washed with PBS supplemented with 5% inactivated FBS, to obtain cells. The cells were suspended in differentiation medium DMEM (supplemented with 30% of supernatant from the L929 cell culture, 20% of FBS, 1% of L-glutamine and 100 UI/mL of penicilin G), distributed in Petri dishes and incubated at 37°C with 5% CO_2._

At the 4th and 7th days after bone marrow removal, more differentiation medium was added to the Petri dishes. At the 9th day of cultivation, the supernatant was aspirated and discarded. Each plate received 5 mL of a sterile solution of ethylenediamine tetraacetic acid (EDTA) 20 mM (Synth, Diadema, SP, Brazil) and was subsequently incubated at 37°C with 5% CO_2_ for 20 min. At the end of the incubation period, 5 mL of FBS were added to each Petri dish. Cell scrappers were used to detach the cells. The subsequent cellular suspensions were transferred to sterile conical bottom tubes and washed with PBS. Concentrations were adjusted to 1 × 10^6^ cells/mL in CTCM and final volumes of 2 mL (at 2 × 10^6^ cells per well) were plated in polystyrene plates. An average of 30–33% of BMMΦ was obtained after the differentiation process.

#### BMMΦ Infection by R0 or R60

BMMΦ were infected with 2 × 10^6^ promastigotes of either R0 or R60, grown for 7 days (late stationary phase) in axenic cultures. The parasites were centrifuged at 50 g for 10 min; dead parasites were discarded, and the viable ones were added to the BMMΦ culture in an average proportion of five parasites per BMMΦ. After 2 h of homogenization, the cells were washed with sterile PBS to eliminate the non-internalized parasites (time 0 h). The wells were analyzed after 3, 12, and 24 h (times 3, 12, and 24 h, respectively).

To evaluate *in vitro* infectivity, 8 × 10^5^ cells/mL were plated in chamber slides (*Lab-tek chamber slides*, NUNC, Thermo Fisher Scientific). Panotic (Laborclin, Pinhais, PR, Brazil) was used to stain slides, following manufacturer instructions. Olympus BX50 optical microscopes *(Olympus, Center Valley, PA, USA)* were used for microscopic examination of slides to determine BMMΦ infectivity. A minimum of 300 BMMΦ per chamber well was analyzed for each SIVP cultures and for each time interval, and the numbers of infected and non-infected cells were determined. The experiment was performed in triplicate. Two-way ANOVA was used for statistical analyses.

### Enzyme Linked Immunosorbent Assay (ELISA)

Spleens obtained after 2 weeks of infection with either R0 or R60 were collected and homogenized. The obtained suspensions had their concentrations adjusted to 5 × 10^6^ cells/mL (CTCM, pH 7.2). The cells were distributed into 48 well plates and stimulated with 50 μg/mL of homolog *Leishmania* particulate antigen. 10 μg/mL of concanavalin A (ConA) was used as a positive control, while the CTCM with no stimulus was used as a negative control (Coelho et al., 2003). The plates were incubated for 48 h at 37°C with 5% CO_2_ and then centrifuged at 706 g at room temperature for 5 min. Supernatants were collected and stored at −20°C for later cytokines assays.

Quantification of cytokines in culture supernatants was performed by enzyme-linked immune sorbent assay (ELISA). BD OptEIA (BD Biosciences, San Diego, CA, USA) kits were used for measurement of IFN-γ and IL-10, following the manufacturers' guidelines.

### Metabolite Extraction for Metabolomic Fingerprint Analyses of Promastigotes

For each group, 10 biological replicates were collected at the 4th day of cultivation. Before extracting the metabolites, the culture bottle (10 mL volume) was shaken in an ethanol/dry-ice bath for 40 seconds for quenching. For UPLC-MS and CE-MS, 4 × 10^7^ promastigotes were centrifuged at 2,000 g for 10 min at 4°C, washed 3 times in cold (4°C) PBS and lysed in 450 μL of cold (4°C) CH_3_OH/H_2_O (4:1, v/v). The cells were mechanically disrupted by 3 cycles of freeze/thaw in liquid N_2_ and then by lysing for 10 min at 50 Hz in a TissueLyser LT (Qiagen) with glass beads (50 mg, 425–600 μm, Sigma-Aldrich). Cellular debris were removed by centrifugation at 15,700 g at 4°C for 10 min. For UPLC-MS, 200 μL of clarified supernatants were transferred to a glass vial and analyzed. For CE-MS, 200 μL of clarified supernatants were transferred to a new tube, dried, resuspended in 200 μL of formic acid (0.1 mmol/L) containing methionine sulphone (0.2 mmol/L–internal standard), vortex mixed for 1 min and then centrifuged at 15,700 g at 4°C for 10 min.

For GC-MS, the metabolites were extracted using 350 μL of CH_3_OH/CHCl_3_/H_2_O (3:1:1, v/v/v) at 4°C, lysed for 10 min at 50 Hz in a TissueLyser LT (Qiagen) with glass beads (50 mg, 425–600 μm, Sigma-Aldrich). The supernatants (200 μL) were clarified by centrifugation and evaporated until dry in a SpeedVac at 30°C. Afterwards, 10 μL of O-methoxyamine hydrochloride (15 mg/mL in pyridine) was added to each vial, mixed vigorously for 5 min with a vortex FB 15024 (Fisher Scientific, Spain) and incubated in darkness at room temperature for 16 h for methoximation. Later, 10 μL of BSTFA [N,O-bis(trimethylsilyl)trifluoroacetamida] with 1% TMCS (v/v) (trimethylchlorosilane) was added, the vials were vortexed for 5 min and incubated for 1 h at 70°C for the silylation reaction. Finally, 100 μL of 10 mg/mL of C18:0 methyl ester in heptane (internal standard) were added and the samples were vortexed. Two blank samples were prepared following the same procedures of extraction and derivatization.

Quality controls (QCs) were independently prepared for each technique by pooling equal volumes of each sample. They were evaluated at the beginning of each analysis to reach system equilibration and throughout each run to provide a measurement of the system's stability as well as the reproducibility of sample treatment procedure (Canuto et al., 2014).

### Metabolomic Fingerprinting by UPLC-MS, CE-MS, and GC-MS

Samples were analyzed by UPLC-MS, CE-MS, and GC-MS, to ensure a wide coverage that would encompass hydrophobic, hydrophilic, acid, basic and neutral molecules. CE-MS and GC-MS were performed according to the methods previously described by Canuto et al. (2014). UPLC-MS was performed according to the methods previously described by Bujak et al. (2014) and Canuto et al. (2014).

### Metabolomic Data Treatment and Statistical Analyses

The resulting data files (UPLC-MS and CE-MS) were cleaned of background noise and unrelated ions using the Molecular Feature Extraction (MFE) tool in the Mass Hunter Qualitative Analysis software (B.05.00, Agilent). Primary data treatment (filtering and alignment) was performed using the Mass Profiler Professional software (B.02.01, Agilent). Data treatment for the GC-MS analysis was conducted by using the Fiehn retention time locked (RTL) Library and the National Institute of Standards and Technology mass spectra library (Kind et al., 2009; Canuto et al., 2014), with the MSD ChemStation software (G1701EA E.02.00.493, Agilent) and a correct assignment based on the coincidence of retention times and spectrum profiles (Canuto et al., 2014). Features that did not appear in at least 50% of the QCs with a coefficient of variation lower than 30% were excluded from analysis in all analytical platforms. The Heatmap (Figure 4B) was constructed with R software (R x64 3.1.3), using gplots packages and Heatmap2 script. The clValid package with K-means script was applied to cluster metabolites into groups. The MBRole *on line* software (http://csbg.cnb.csic.es/mbrole/) was used to map pathways and the biological role of compounds.

The filtered masses were exported to SIMCA-P+ 12.0 (Umetrics) for multivariate statistical analysis. To approximate to a normal distribution, data were converted to the logarithmic scale before any statistical calculations (Canuto et al., 2014). To check for quality, principal component analysis (PCA-X) models were used to visualize QC clustering with the non-filtered data. Later, partial least square discriminant analysis (PLS-DA) models were built to discriminate the samples in the groups. The quality of these models was described by explained variance (R^2^) and predicted variance (Q^2^) the former explains variance and fitness, while the latter predicts variance and provides information about model predictability. The predictive ability of the multivariate models was confirmed in accordance to the orthogonal projection to latent structure discriminant analysis (OPLS-DA) (Canuto et al., 2014). Both PLS-DA and OPLS-DA were built using the filtered data. Unit variance (UV) scaling and logarithmic transformation (base 2) were used.

For LC-MS, GC-MS, and CE-MS data, differences among R0xR10, R0xR40, and R0xR60 were evaluated for individual metabolites using univariate statistical analysis (*t*-test for normal distribution or Mann-Whitney *U* test when the variable did not follow a normal distribution), using in-house algorithms in MATLAB, version 7.10 (MathWorks Inc., Natick, Massachusetts). Fold changes were calculated by averaging the abundance results between groups in Log_2_.

Accurate masses of features representing significant differences (*p* < 0.05 in at least two statistical analyses) obtained from MS-based analyses were searched against the CEU mass mediator database (http://biolab.uspceu.com/mediator/). For each statistically significant mass, the formula proposed by the MassHunter software (B.05.00, Agilent Technologies) was compared with the experimental isotopic pattern distribution.

For CE-MS data, commercial standards, when available, were used to confirm putative identifications, combining peaks of samples and peaks of standards at the same run. Confirmation of matches was performed by comparison of the migration time and isotopic distribution.

### Evaluation of Cellular Oxidative Stress During SIVP Process

To evaluate the cellular oxidative stress, promastigotes from R0 and R60 were cultivated in M199 medium (Gibco) supplemented with 10% of fetal bovine serum (Gibco) and cultivated at 26°C. R0 and R60 cultures at late log (4th day) phase were used. Aliquots containing 10^6^ parasites were centrifuged at 1,540 xg and washed with PBS twice. The cultures were treated as recommended by manufacturer of CellROX^®^ Deep Red Reagent (Thermo Fisher Scientific). The levels of ROS were measured by flow cytometry using BD Fortessa. Experiments were performed in triplicates. The statistical analyses and graph plots were performed using GraphPad Prism 8.0.1

### Susceptibility Assay of *L. amazonensis* SIVP Parasites to Antimony (Sb^III^)

*L. amazonensis* (R0, R10, R40, and R60) promastigotes at the 4th day of cultivation were submitted to Sb^III^ (Sigma-Aldrich, St. Louis, MO, USA) susceptibility tests. Suspensions of parasites at 2 × 10^6^ cells/mL were incubated in 24-well plates with M199 medium at 26°C for 48 h. The parasites were incubated either in the absence of Sb^III^ or exposed to several different concentrations of that compound (18.7–374.3 μM). The effective concentrations required to kill 50% of the parasites (EC50) were determined using a model *Z1 Coulter Counter* (Beckman Coulter, Fullerton, CA, USA). EC50 values were determined from at least two independent measurements performed in triplicate, applying the method of linear interpolation (Huber and Koella, 1993). Statistical analyses were performed using GraphPad Prism Software v5.0, San Diego, CA.

### Measurement of Phosphatidylserine Exposure by *L. amazonensis* SIVP Promastigotes Using FACS

The BD AnnexinV-PE Apoptosis Detection Kit was used to determine of aPS exposure by *L. amazonensis* submitted to SIVP. The protocol described by Rochael et al. (2013), with modifications was used. Suspensions of 5 × 10^5^ cells/mL of promastigotes (R0, R10, R40, and R60) at the 4th and 7th days of growth were collected by centrifugation at 1,450 g, for 10 min at 4°C, washed twice in PBS and suspended in 100 μL of binding buffer (10 mM HEPES [pH 7.4], 150 mM NaCl, 5 mM KCl, 1 mM MgCl_2_, and 1.8 mM CaCl_2_). To each cell suspension, 2 μl of BD AnnexinV-PE and 2 μl of 7-Amino-Actinomycin (7-AAD) were added. No-labeled suspensions were used as negative controls, while positive controls were defined as those labeled either with AnnexinV-PE only or 7AAD only. The suspensions were incubated for 15 min in the dark, at room temperature. Triplicate analyses were performed for each SIVP. 7-AAD was used to determinate parasite viability, while AnnexinV-PE was used to measure aPS exposure. Graphs with PerCP-Cy5.5 (7-AAD) in the y axis and PE (annexinV) in the x axis were constructed, and gates were determined as following: lower left gates for no-labeled parasites; upper left gates for 7-AAD (dead parasites); upper right gates for 7-AAD+ annexinV (non-viable, aPS-exposing parasites); and finally lower right gates for AnnexinV-PE (viable, aPS-exposing parasites). Thus, the percentages of aPS exposure were calculated considering the number of parasites in the lower right gates (Supplemental Figure 5).

Results were obtained by flow cytometry, using a BD LSRFortessa™ cytometer. BD FACSDiva™ software was used for acquisition and analysis. Results were analyzed by FlowJo^®^ V10. Statistical analyses were performed using GraphPad Prism Software v5.0, San Diego; CA. Two-way ANOVA was used for multiple group comparisons. Statistically significant values were defined as *p* < 0.05.

### RNA Extraction and cDNA Synthesis

R0, R10, R40, and R60 promastigotes were cultivated in 20 mL of M199 medium until the 4th day of growth, as previously described. Cell suspensions were centrifuged at 5,000 g at 4°C for 10 min, using DNA/RNA-free tubes with 1.5 mL of sterile water. The parasites were suspended in 500 μL of RNA Later (Sigma-Aldrich, MO, USA) and stored at −80°C for further processing. When later processed, the suspensions were thawed and centrifuged at 5,000 g at 4°C for 10 min. RNA-Later supernatants were discarded. Total RNA was extracted with Tri-Reagent (Sigma-Aldrich, MO, USA), following the manufacturer's instructions. Quality, concentration and purity of the extracted RNA were measured by 1.5% agarose gel, Qubit 2.0 (Thermo) and NanoDrop (Thermo), respectively. Total RNA was treated with DNaseI (DNase I, RNase-free 1 U/μL, Thermo Fisher) following the manufacturer's instructions. cDNA was synthesized from 1 μg of total RNA with the High-Capacity cDNA kit (Thermo Fisher), following the manufacturer's instructions.

### qPCR Analyses

Selected loci (Supplemental Table 1) were amplified using the Power Syber Green kit (Thermo Fisher) with 0.2 μM primers. The GAPDH locus was used as reference to normalized gene expression, while the R0 group was used as control. Quantitative PRC reactions were performed on the QuantiStudio 3 thermocycler (Thermo Fisher) with the following program: 50°C for 2 min, 95°C for 10 min, 40 cycles of 95°C for 15 s and 60°C for 1 min, followed by a dissociation curve of 60°C to 95°C (0.1°C/s). The values of Ct for each locus and each group were evaluated based on three different experiments. The expression levels were calculated using the ΔΔCT, as previously described (Pfaffl, 2001). Statistical analyses were performed using Two-way ANOVA with Bonferroni post-test.

## Results

### *L. amazonensis* Parasites Resultant From SIVP Are Less Infective to Mice

In a previous study (Magalhães et al., 2014), the *L. amazonensis* PH-8 strain, recently isolated from mice (R0), was submitted to successive *in vitro* passages (SIVP) in axenic culture, generating the R10 and R30 parasites (obtained after 10 and 30 SIVP, respectively). Here, the R0 and R10 SIVP cultures, in addition to R40 and R60, also derived from R0, were further characterized through a metabolomics fingerprinting approach.

**Figure 1:**
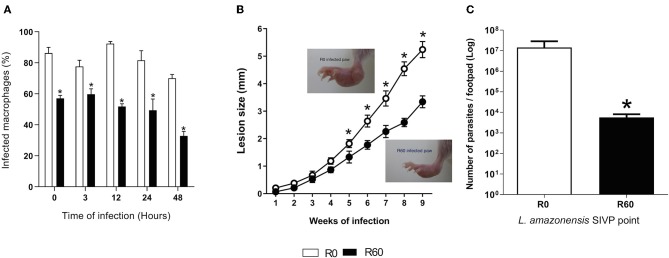
*In vitro* BMMΦ infection, lesion size development and tissue parasitism in footpads of BALB/c infected with R0 and R60 strains. **(A)** Percentage of infected bone marrow-derived macrophages. BALB/c bone marrow-derived macrophages (2 × 10^5^ cells/ml) were infected with R0 or R60 grown for 7 days, at late stationary phase (5 parasites/macrophage). The cells were fixed and stained with Giemsa after 3 h of exposure (defined as 0 h time) and after 3, 12, 24, and 48 h post infection. The percentage (%) of infected macrophages was obtained by manual counting using an optical microscope. 300 cells at three different wells were evaluated from two different assays. **(B)** Evaluation of footpad lesion size from BALB/c mice infected with 1 × 10^6^ *L. amazonensis* R0 or R60 stationary phase promastigotes. Lesion size was weekly measured with a caliper, and represent the size of infected paw minus the size of the non-infected one. Each point represents an average of lesion size from six infected mice plus the standard error (95% confidence interval). In detail, photographs of R0 and R60 infected paws at 8 weeks post-infection. **(C)** Limiting dilution assay, performed at 2nd week post-infection. Infected BALB/c footpads were homogenized in a glass homogenizer with M199 medium. The obtained pellet was resuspended in 500 μl of M199 medium and submitted to serial dilution (in duplicate) 1:10 in 96-well micro plates at final volume of 200 μL and incubated at 26°C for 15 days. The last dilution at which viable parasites was observed was considered the final titer. Results were expressed as negative logarithm of the parasites title. Bars represent mean values of three biological replicates from four infected BALB/c footpads plus standard deviation of the mean. ^*^ indicates *p* ≤ 0.05 (Two-way ANOVA with Bonferroni post-test).

Initially, to validate that the SIVP process decreased infectivity, late stationary phase promastigotes (7th day of axenic growth) of R0 and R60 were used for *in vitro* infection of BALB/c mice bone marrow-derived macrophages (BMMΦ). As shown in Figure 1A, the percentages of BMMΦ infected with R60 were significantly lower as compared to R0, at all time points post-infection, indicating that R60 was less infective than R0. To determine if SIVP also diminished infectivity *in vivo*, BALB/c mice were infected at the right hind footpad with R0 and R60 and lesion development was monitored weekly. Differences in lesion size induced by R0 and R60 in BALB/c footpads were detected after 5 weeks of infection. Footpad swelling was significantly smaller in mice infected with R60, when compared to those infected with R0 (Figure 1B). After 2 weeks of infection, parasite loads (detected by limiting dilution assays) were also lower on BALB/c mice infected with R60 when compared to those infected with R0 (Figure 1C). These findings confirmed that, as expected, R60 promastigotes have impaired infectivity at the initial stages of infection in BALB/c mice.

### Long-Term *in vitro* Cultivation Does Not Affect Growth Rates and the Promastigote Metacyclogenesis Process

To verify if SIVP interfered in the capacity of proliferation of the SIVP parasites, growth curves were evaluated (Supplemental Figure 1). R10, R40, and R60 displayed the same growth rates when compared to R0, indicating that, under the same conditions, SIVP did not impact on parasite growth rates.

**Figure 2:**
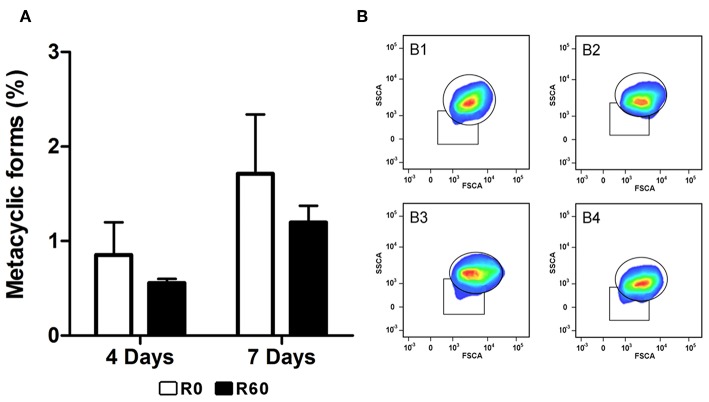
Evaluation of metacyclogenesis in R0 and R60 cell lines. Promastigotes from R0 and R60 cell lines were cultivated in M199 medium (10% BFS, 26°C). R0 and R60 cultures at late log (4th day of growth) and stationary phases (7th day of growth) were analyzed by flow cytometry using BD Fortessa. **(A)** Percentages of promastigote metacyclic forms in R0 and R60 cell lines in late log (4 days) and stationary (7 days) phases. **(B)** B1 and B2 represent, respectively, R0 and R60 at the 4th day of growth. B3 and B4 represent, respectively, R0 and R60 at the 7th day of growth. The bars represent the average of two assays, minus and plus the standard deviation, in two independent analyses. Significant differences were investigated by Two-Way ANOVA.

Since metacyclogenesis is an important factor that affects *Leishmania* spp. infectivity and might be altered by long-term axenic growth (da Silva and Sacks, 1987; Cysne-Finkelstein et al., 1998), R0 and R60 promastigotes were also compared for their ability to differentiate *in vitro* from procyclic to metacyclic promastigote forms. Initially, enriched populations of procyclic and metacyclic were analyzed by a flow cytometry assay (FACS) to determine the gates that corresponded to each cell type (Supplemental Figure 2). FACS analysis was chosen because it allows discrimination of cells according to granularity and cellular volume and (FCS), and it is known that procyclics and metacyclics differ in size (Spath and Beverley, 2001; da Silva et al., 2015). Later R0 and R60 promastigotes were submitted to FACS analysis at the 4th and 7th days of cultivation (late log and stationary phases, respectively) (Figures 2A,B). The gates previously determined were used to indicate the populations that displayed low size and granularity. No significant differences were observed between R0 and R60 cultures at the analyzed time points, suggesting that the decreased infectivity of R60 is not associated with a decreased ability to generate metacyclics.

### Infection With the Attenuated *L. amazonensis* R60 Promastigotes Is Associated With Increased Levels of Specific IFN-γ During the Early Stages of Infection in BALB/c Mice

**Figure 3:**
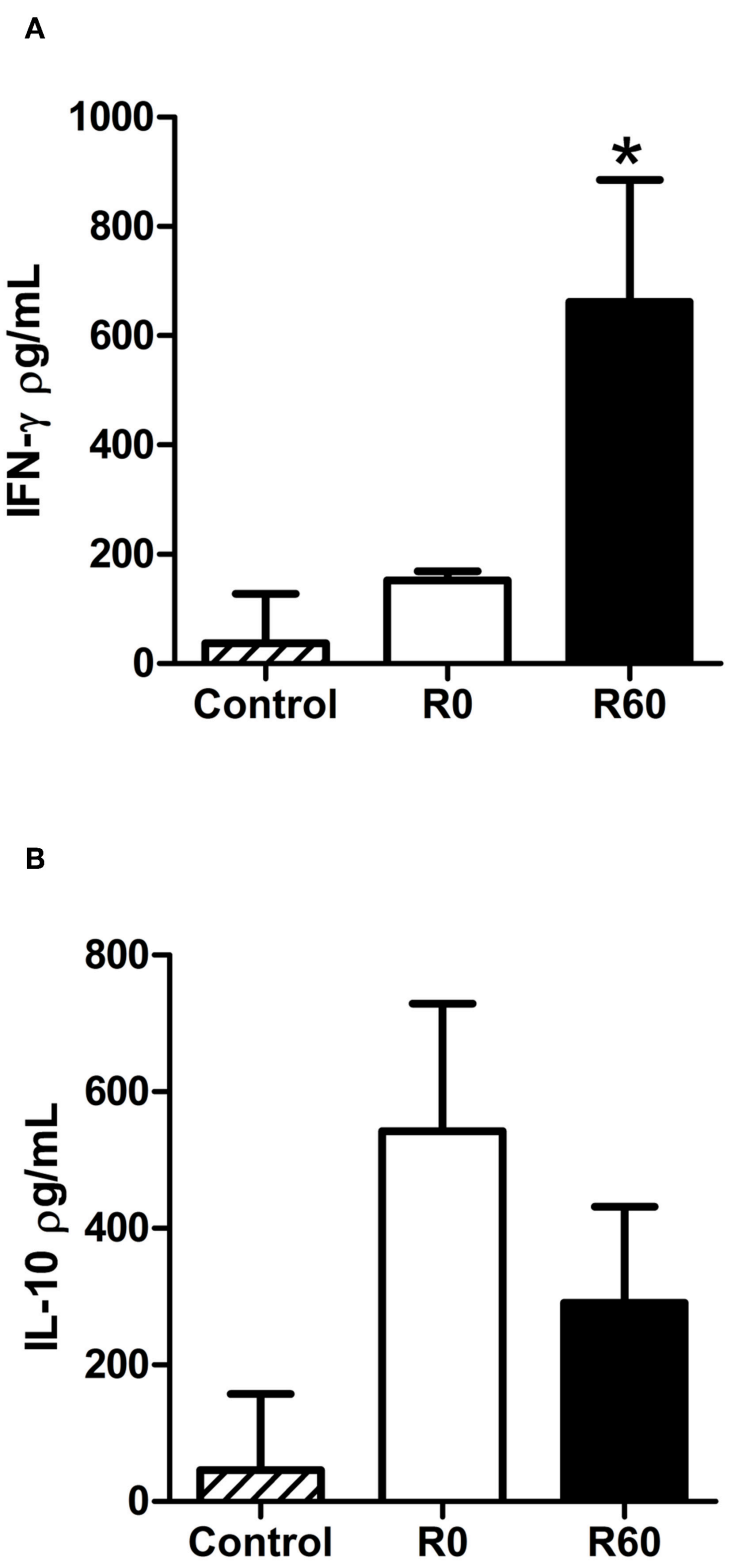
Comparison of cytokine levels in spleen culture supernatants from BALB/c infected with R0 and R60 cell lines after 2 weeks of infection. For infection, 1 × 10^6^ parasites were inoculated at the right footpad. Two weeks later, the animals were euthanized and RPMI medium (34°C, 5% C0_2_) was used to cultivate spleen cells. The cultures were stimulated with soluble *Leishmania* antigen (SLA). Negative controls were non-stimulated spleen cells. 48 h after the stimulation, supernatant was collected and an ELISA assay was performed. **(A)** Levels of specific IFN-γ observed in spleen culture supernatants from BALB/c infected with R60 as compared to the levels produced by cells from mice infected with R0. **(B)** IL-10 levels in supernatant of splenocytes from R0 and R60. Bars represent mean values of three biological replicates from four infected BALB/c footpads, plus standard deviation of the mean. ^*^indicates *p* ≤ 0.05 (Two-way ANOVA with Bonferroni post-test).

Host immune responses modulate the course of *Leishmania* infection, while also reflecting its inflammatory responses to infection (Carneiro et al., 2015; Maspi et al., 2016; Conceicao-Silva et al., 2018). As previously shown, infection by R60 induced both smaller lesions and parasite loads in BALB/c mice, in relation to R0 promastigotes. To investigate if this profile could be associated to alterations in host immune responses, the levels of specific IFN-γ and IL-10, key cytokines enrolled, respectively in resistance and susceptibility to *Leishmania* infections, were measured in supernatants of splenocytes culture from BALB/c mice infected with R0 and R60. Two weeks post-infection, increased levels of IFN-γ were detected in spleen culture supernatants of mice infected with R60, when compared to those from R0 infected mice, after stimulation with promastigotes total protein extracts (Figure 3A). However, no significant differences were seen in IL-10 levels (Figure 3B).

### The Metabolomic Multiplatform Fingerprint Approach Revealed the Biochemical Pathways Involved in *L. amazonensis'* Attenuation

To uncover the continuous metabolic changes occurring during SIVP, a multiplatform metabolomic fingerprint approach (GC-MS, LC-MS and CE-MS) was applied to obtain the metabolic profile of late log-phase (4th day of growth) promastigotes' extracts of R0, R10, R40, and R60, grown under the same conditions. QC samples, prepared by pooling aliquots of all samples, were used to monitor the entire analytical processes. These QC samples were applied recurrently at different intervals to monitor equipment performance.

The obtained metabolomics data were first filtered to exclude metabolites that presented extreme variations: more than 30% of quality controls (QC, %RSD) and in addition, those that were not present in at least 80% in each group (filtered by flags). Then, a PCA-x analysis was applied to the filtered features. The QC samples were very tightly clustered for each analytical method, indicating a precise analytical performance and high experimental reproducibility (Supplemental Figure 3). Subsequently, a supervised partial least squares discriminant analysis (PLS-DA) was performed. Samples from different SIVP promastigotes were clustered in well-defined groups, as a first indication of significant metabolic differences among the SIVP promastigotes (Supplemental Figure 4).

The chemometric analysis revealed significant changes in ionic abundance in 66 metabolites that could be associated to *L. amazonensis* SIVP. Of these, 30 metabolites were detected by GC-MS and had their identities confirmed by fragmentation pattern, using the Fiehn RTL Library (Agilent) (Kind et al., 2009); 17 compounds were putatively identified by LC-MS analysis in both positive and negative modes, while 19 compounds were identified using CE-MS; 10 compounds were confirmed by comparison with samples spiking off the corresponding standards (Naz et al., 2014), while nine were putative. Detailed results, including retention time (RT), theoretical and experimental compounds' mass, mass error in ppm and %RSD are shown in Supplemental Table 2.

**Figure 4:**
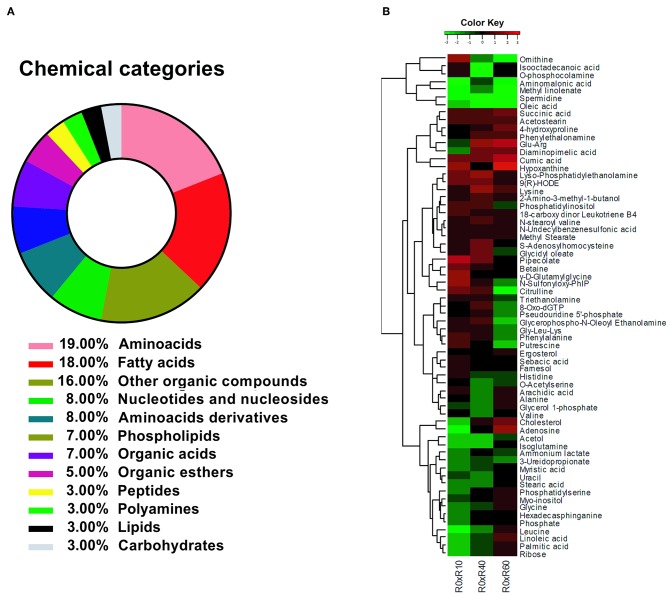
Identification of *L. amazonensis* metabolites' changes during SIVP. **(A)** Biochemical categories for the identified metabolites in *L. amazonensis* attenuation process after SIVP, identified by multiplatform metabolomic fingerprint approach. Metabolites were classified according to their chemical category by MBRole online software. **(B)** Heatmap showing confirmed or putatively identified metabolites in log2-scale fold change. To obtain a Kinect metabolite profile, two intermediary strains were included in this analysis, R10 and R40 (10 and 40 SIVP, respectively). The dendogram shows the fold changes behavior for each metabolite among all metabolites (metabolome), except for Proline, which extrapolated the fold change for the majority of the metabolites and did not fit into the scale. Metabolites' fold changes were calculated by comparing the ionic abundance average (in Log_2_) from each metabolite belonging from each attenuated lines (R10, R40, and R60) to the virulent one (R0). The Values varied from −3.0 (green) to 3.0 (red). Non-expressive fold changes (values next to zero) were shown in black. The heatmap were generated by the R x 64 3.1.3 software, using the gplots/heatmap2 package.

The identified metabolites were, afterwards, grouped according to their chemical categories: 27% were classified as amino acids and amino acids derivatives, 18% as fatty acids, 16% as other organic compounds, 8% as nucleotides and nucleosides, 7% as phospholipids, 7% as organic acids, 5% as organic esters, 3% peptides, 3% as polyamines, 3% as lipids, and 3% as carbohydrates (Figure 4A). Altogether, 33% of the identified compounds were associated to fatty acids metabolism.

By comparing R0 with each of the other SIVP promastigotes, the fold changes of 65 metabolites' levels (except for proline) were hierarchically clustered and graphically represented in a heatmap (Figure 4B). Proline displayed a significant fold change increased throughout SIVP, starting from −1 and raising to 3.52 at R40 and 5.22 at R60, being far out of the heatmap scale for most of the other compounds, and thus was not represented in the heatmap. The HCA analysis clustered the metabolites in seven major clusters, according to the pattern of changes throughout SIVP (Figure 4B). Examination of this heat map, reveals that abundance of several metabolites significantly changed throughout the attenuation process, even as early as after 10 SIVP (R0xR10), suggesting that adaptation to this environmental change is a dynamic process and that metabolic alterations are continuously occurring, even after 40 SIVP.

**Figure 5:**
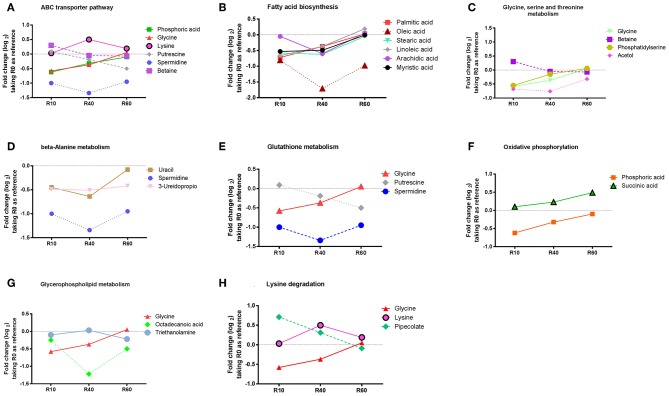
Enriched and non-enriched pathways, identified by MBRole online software, form *L. amazonensis* metabolites associated with the attenuation process, as detected by the metabolomic fingerprint multiplatform approach. Identified metabolites were submitted to MBRole online software, using their KEGG IDs, with *L*. major pre-compiled reference, in order to map those biological pathways with statistic significance. Fifty-two metabolites could be identified with KEGG IDs and were mapped in 42 pathways. Eight pathways were mapped as enriched (statistic significant): ABC transporters **(A)**, Fatty acids Biosynthesis **(B)**, Glycine, serine and threonine metabolism **(C)**, B- beta-Alanine metabolism **(D)**, Glutathione metabolism **(E)**, Oxidative phosphorylation **(F)**, Glycerophospholipid metabolism **(G)**, Lysine degradation **(H)**. The metabolites responsible for identification of each enriched pathways are represented in graphics, showing Log_2_ fold change, expressed as the average ionic abundance in R0xR10, R0xR40, and R0xR60, to obtain a kinetic analysis of metabolites during attenuation by SIVP.

Additionally, an enrichment analysis was performed, using the MBRole software (http://csbg.cnb.csic.es/mbrole/.) Of the 66 identified metabolites, 52 presented KEGG IDs and were used to map eight enriched metabolic pathways. The metabolites used by MBRole to identify the enriched pathways were plotted in individual graphs, as shown in Figure 5: ABC transporters (Figure 5A); fatty acid biosynthesis (Figure 5B); glycine, serine and threonine metabolism (Figure 5C); β-alanine metabolism (Figure 5D); glutathione metabolism (Figure 5E); oxidative phosphorylation (Figure 5F); glycerophospholipid metabolism (Figure 5G) and lysine degradation (Figure 5H). Taken as s whole, the metabolite's abundance decreased, except for lysine and succinic acid, which presented increased levels from R0 to R60 (Figures 5A,F,H). Some metabolites such as betaine, putrescine, and pipecolate showed increased levels until the 10th passage, but significant decreases were further observed in additional stages of axenic cultivation (Figures 5A,C,E). Other metabolites such as glycine, uracil, phosphate, myristic acid, stearic acid, palmitic acid, linoleic acid and arachidic acid showed decreased levels until the 40th passage, but their levels raised to those present in R0, in R60 (All Figure 5, except Figure 5B). Remarkably, phosphatidylserine (PS) was detected in low levels until the 10th passage but increased in further passages (Figure 5C).

### Phosphatidylserine Analogs (aPS) Exposure by SIVP Promastigotes, as Revealed by Flow Cytometry Analysis

**Figure 6:**
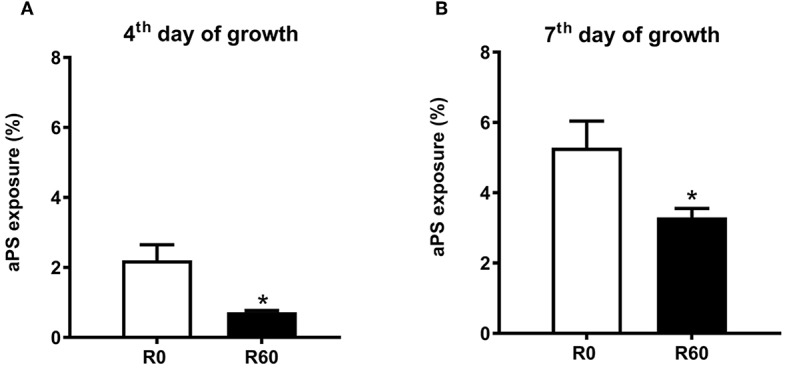
aPs exposure by R0 and R60 promastigotes. **(A)** Profile of promastigotes grown for 4 days. **(B)** Profile of promastigotes grown for 7 days. 2 × 10^5^ parasites from R0 and R60 were washed with PBS, suspended in binding buffer (BD-Anexin V-PE), and then incubated at room temperature for 15 min. At the moment of acquisition, 7AAD (PerCP-Cy5.5-A) was added as viability control, following manufacturer instructions. Data is shown as the difference between the geometric mean of the fluorescence intensity of unstained control samples (Supplemental Figure 5—Q1) compared to annexin V(PE) stained ones (Supplemental Figure 5—Q4). Viability was checked by 7AAD (PerCP-Cy5.5-A) parasites labeled (Supplemental Figure 5—Q2 and Q3). Data were collected in BD Fortessa and analyzed by FlowJo-X. At least 10,000 gated events were collected from each sample, in triplicates, from two essays. ^*^p ≤ 0.05 Two-Way ANOVA.

The metabolomic analysis revealed concomitant changes in the abundance of various lipids and glycerophospholipids, suggesting significant remodeling on membrane lipid composition during SIVP. Among glycerophospholipids, the increased exposure of analogs of phosphatidylserine (aPS) has been reported as important for *Leishmania* virulence (Franca-Costa et al., 2012; Rochael et al., 2013). There was a significant increase in the intracellular contents of PS in R40 and R60 parasites. However, R60 was less virulent than R0. This finding prompted the hypothesis that R60 exposes less aPS than R0. In order to confirm that aPS metabolism changed in attenuated parasites, we measured aPS exposure by flow cytometry (Franca-Costa et al., 2012; Rochael et al., 2013). The gates for aPS analysis were determined using non-labeled parasites (Supplemental Figure 5A, Q1), aPS labeled with only annexinV (PE) (Supplemental Figure 5B, Q4) and dead parasites labeled only with 7AAD (PerCP-Cy5.5) (Supplemental Figure 5C, Q2). Significant differences were observed in aPS exposure between R0 and R60, both at the 4th and 7th days of axenic growth (Figures 6A,B). Overall, lower aPS levels were presented by R60 than R0. This finding, in conjunction to the metabolomic data, may suggest that aPS are being accumulated in the promastigotes' cytoplasm and being less exposed at the cell surface.

### qPCR Analysis of Selected Genes Involved in the Identified Pathways

**Figure 7:**
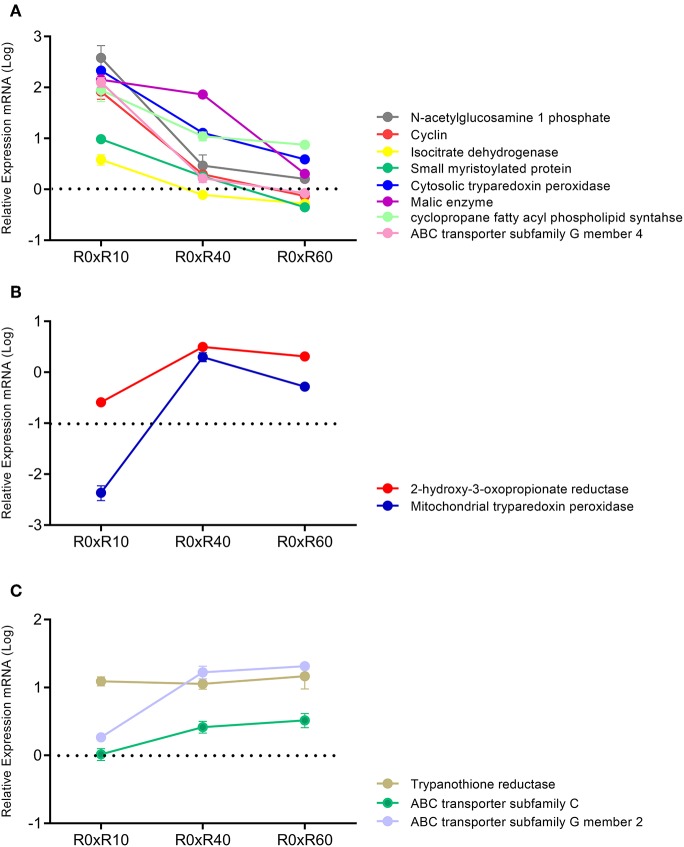
qPCR analysis for expression of 13 genes related to the SIVP process. All measured loci were classified in three clusters, according to its expression. Data represent fold changes for R10, R40, R60, in relation to R0. **(A)** Cluster 1-N-acetylglucosamine 1 phosphate, Cyclin, Isocitrate dehydrogenase, Small myristoylated protein, Cytosolic tryparedoxin peroxidase, Malic enzyme, Cyclopropane fatty acyl phospholipid synthase, ABC transporter subfamily G member 4. **(B)** Cluster 2-2-hydroxy-3-oxopropionate reductase, Mitochondrial tryparedoxin peroxidase. **(C)** Cluster 3-Trypanothione reductase, ABC transporter subfamily C, ABC transporter subfamily G member 2. Samples were analyzed in duplicates, from three different experiments.

Attempting to correlate the previous proteomic data of R0 and R30 (Magalhães et al., 2014) and the herein gathered metabolomic data, qPCR analyses of 13 selected genes linked to the proteins and metabolic pathways involved in attenuation after SIVP was carried out. Expression levels of GAPDH were used to normalize the expression levels of the selected loci. As shown in Figure 7, mRNAs expression profiles were classified in three clusters according to their expression pattern. The first cluster included the genes with increased gene expression in R10 related to R0 (N-acetylglucosamine 1-phosphate, cyclin, isocitrate dehydrogenase, small myristoylated protein, cytosolic tryparedoxin peroxidase, malic enzyme, cyclopropane fatty acyl phospholipid synthase and ABC transporter subfamily G member 4). However, their expression levels decreased progressively during further SIVP (R40 and R60) (Figure 7A). The second cluster, comprised by 2-hydroxy-3-oxopropionate reductase and mitochondrial tryparedoxin peroxidase, was characterized by decreased expression in R10 as compared to R0, and further increase, reaching levels similar to R0, in R40 and R60 (Figure 7B). The last cluster encompassed genes with increased expression during the entire SIVP process: ABC transporter subfamily G member 2, trypanothione reductase and ABC transporter subfamily C (Figure 7C).

### SIVP Parasites Produced Increased Levels of ROS and Are More Sensitive to Antimony Tartrate

**Figure 8:**
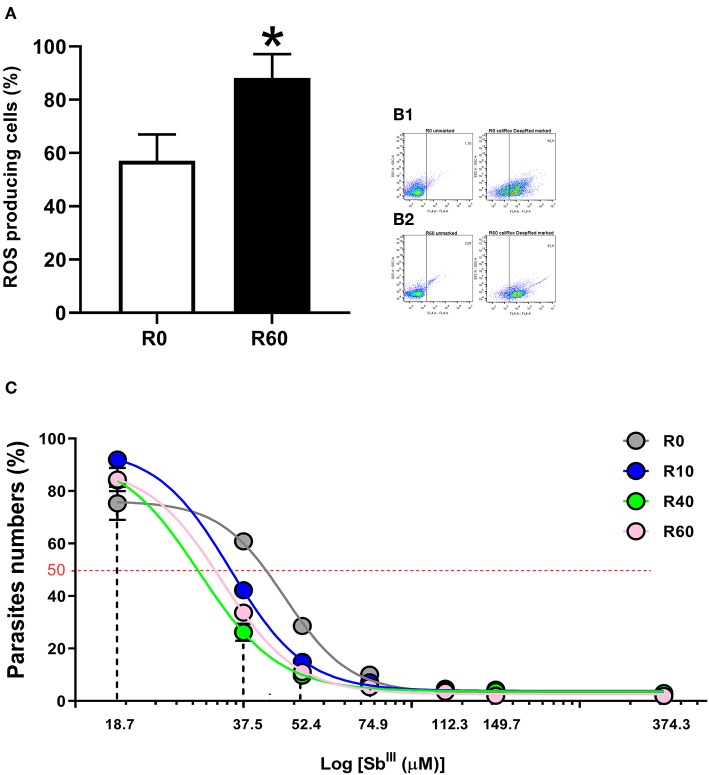
Reactive oxygen species (ROS) measurement in *L. amazonensis* promastigotes after SIVP. R0 and R60 promastigotes were cultivated in M199 medium (10% BFS, 26°C). The level of ROS was assessed by CellROX Deep Red Reagent (Thermo Fisher Scientific). After treatment, R0 and R60 cultures at late log phase (4th day) were analyzed by flow cytometry (BD Fortessa). **(A)** Percentage of cells producing ROS from R0 and R60. **(B1,B2)** Gates used to determine the percentage of R0 and R60 ROS producing cells, respectively. The bars represent the average of tree assays minus or plus the standard deviation from two independent tests. **(C)** Cytotoxic effects of trivalent tartrate antimony (Sb^III^) on *L. amazonensis* (R0, R10, R40, and R60) promastigotes at the 4th day of cultivation. Parasites were incubated in M199 medium at 2 × 10^6^ cells/mL into 24-well plates (26°C), either in the absence or presence of several concentrations of Sb^III^ (18.71–374.32μM) for 48 h. The effective concentration required to decrease growth by 50% (EC_50_) was determined using a Z1 Coulter Counter (Beckman Coulter, Fullerton, CA, USA). EC_50_ values were determined from at least three independent measurements performed in triplicate, using the linear interpolation method. Statistical analyses were performed using GraphPad Prism Software v6.0, San Diego, CA. The EC_50_ values were 47.09, 35.05, 29.52, 33.34 (μM; R0–R60, respectively; R2-values were >0.95 for all cell lines). ^*^p ≤ 0.05 by Two-Way ANOVA.

Energy metabolism, reactive oxygen species (ROS) production and cell detoxification pathways are tightly balanced. Shifts in this balance enable ROS to activate intracellular signaling and/or induce cellular damage and cell death (Liemburg-Apers et al., 2015). Since alterations in energetic metabolism and cell redox pathways were concomitantly by proteomics and metabolomics of SIVP parasites, we investigated if ROS levels were significantly altered in SIVP parasites, using a very specific reagent (CellRox deep red) that becomes oxidized and fluorescent when ROS is present. As shown in Figure 8A, R60 parasites displayed significantly increased levels of cell ROS, when compared to R0. Figure 8B show how gates were determined for this analysis.

Besides energy and glutathione metabolism, metabolomic, proteomic and qPCR data also pointed out significant alterations in the ABC transporters pathway. Since these pathways have been significantly associated to drug efflux and antimonial (Sb^III^) resistance in *Leishmania* (Wyllie et al., 2004; Leprohon et al., 2006), we hypothesized that these alterations in the cellular redox system and cell transport of the SIVP promastigotes may have affected their sensitivity to Sb^III^ tartrate. To test that, Sb^III^ tartrate EC_50_ was comparatively determined for all SIVP parasites. Sb^III^ tartrate ranged from 18.71 to 373.32 μM. As shown in Figure 8C, EC_50_ corresponded to 47.09, 35.05, 29.52, and 33.34 μM for R0, R10, R40, and R60, respectively. Therefore, SIVP promastigotes are significantly (all *R*^2^ > 0.95) more sensitive to trivalent antimony when compared to R0, supporting our hypothesis.

### Merging SIVP Metabolomics and Proteomics Data to Built a Biochemical Map

**Figure 9:**
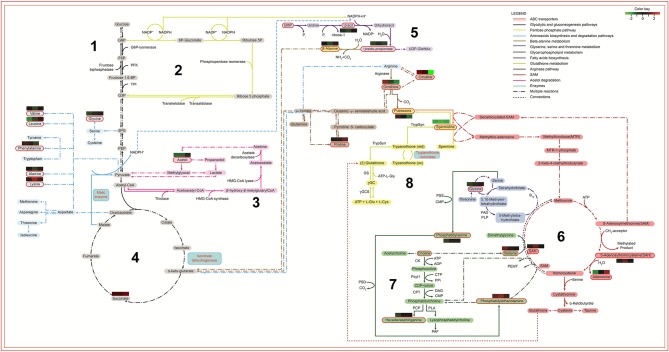
Biochemical map of potential metabolic alterations occurred during axenization of *L. amazonensis*. Metabolites are represented by colored rectangles. Metabolites identified via metabolomic analysis are highlighted with a red contouring. The colored rectangles localized above/under metabolites represent the relative difference of abundance by comparing R10, R40, and R60 to R0 (left to right, respectively). Arrows represent reactions with enzymes, co-enzymes, metabolites or derivatives, indicated by their respective names or initials. Period-dash-period arrows indicate the occurrence of multiple reactions. Small dashes indicate interconnection of metabolic data between different metabolic pathways. The colors of arrows identify their respective pathways: 1. Black for energetic pathways (glycolytic and gluconeogenesis); 2. Yellow-green for pentose shunt (starting in G6P); 3. Light blue for amino acids biosynthesis; 4. Purple for fatty acids biosynthesis (receiving NADPH from pentose shunt and from malic enzyme reaction); 5. Light brown for β-alanine degradation (starting with ureidopropionate and culminating in α-keto-glutarate); 6. Dark brown for arginase and bright yellow for trypanothione (both pathways are connected in the production of putrescine); 7. Red for SAM or AdoMet pathway (interconnected to the Kennedy pathway, producer of phosphatidiletanolamine, phosphatidilcholine and phosphatidilserine); 8. Dark blue for glycine, serine and threonine biosynthesis. G6P, Glucose 6 phosphate; F6P, Fructose 6 phosphate; Fructose 1,6 bp, Fructose 6 bi-phosphate; PFK, phosphofructokinase; TPI, Triose phosphate isomerase; G3P, Glyceraldehyde 3-phosphate; 3PG, 3-phosphoglycerate; PEP, Phosphoenolpyruvate; UMP, Uridine monophosphate; UDP-GlcNac, Uridine diphosphate N- acetylglucosamine; HMG-CoA-Synthase, Hydroxymethylglutaryl-CoA synthase; NADPH, Nicotinamide adenine dinucleotide phosphate; ACP, Acyl carrier protein; TrypSyn, Trypanothione synthase; TrypRed, Trypanothione reductase; Gly, Glycine; Glu, Glutamine; ATP, Adenosine triphosphate; GS, Glutamine synthetase; SAH, S-adenosylhomocysteine; SAM, S-adennosylmethionine; PSS, phosphatidylserine synthase; PSD, Phosphatidylserine decarboxylase; CMP, Cytidine monophosphate; CK, Choline kinase; Pcyt1, Pyruvate carboxylase t1; CPT, Carnitine palmitoyltransferase; PLA, Phospholipase A; CTP, Phosphocholine cytidylyltransferase; ADP, Adenosine diphosphate; DAG, Diacylglycerol; PAF, Platelet-activating fator; PPi, Cytosolic pyrophosphate; PCP, Pyrrolidone carboxyl peptidase.

Finally, the metabolomic and the proteomic (Magalhães et al., 2014) data were merged into the enriched metabolomic pathways to build a comprehensive biochemical map, disclosing the metabolic alterations that occurred after long-term cultivation of *L. amazonensis* promastigotes. In this map (Figure 9), different colors and sequential numbers represent each pathway; metabolites are shown in colored blocks and proteins in blue squares. The identified metabolites were highlighted with red contouring and their fold changes represented by individual heatmaps (R0xR10, R0xR40, and R0xR60) either above or under their names. Proteomic data were represented by a blue square with blue contouring. The ABC transporter pathway was represented by a red double line square, contouring the whole map.

Considering that carbohydrate metabolism was retooled to supply demands from lipid metabolism, the glycolytic and gluconeogenic pathway (1) should be taken as a starting point on this map, followed by pentose phosphate pathway (2), acetol degradation (3) and tricarboxylic acid cycle (4). These pathways generate NADPH, which will be used for lipids biosynthesis (5) fatty acids biosynthesis; (6) SAM or AdoMet pathway, and (7) glycerophospholipids metabolism) and release a large amount of H^+^ ions, culminating in a redox system overload (8) glutathione metabolism). Finally, the ABC transporters pathway were significantly altered and connected to metabolites of other pathways, since they are transmembrane proteins associated to lipid transport, glutathione metabolism and cell detoxification. The overloaded redox system and the lipid alterations make the parasite more vulnerable to ROS and less infective.

## Discussion

Maintenance of *Leishmania* promastigotes under long-term axenic cultivation may cause a wide variety of alterations that affect parasites virulence. This phenotypic variation is highly influenced by the parasites' species and original population, and the environmental conditions, including nutrient availability supplied by the culture medium and the number of passages in axenic culture. Such ability to adapt to environmental conditions and respond to selective pressures may be attributed to *Leishmania* genome plasticity, the clonal replicative structure of *Leishmania* populations, and the dynamic remodeling of its transcriptome and proteome, which will favor the maintenance of phenotypic diversity inside each population, for suscessful replication and perpetuation of the parasite genome (Rougeron et al., 2010; De Pablos et al., 2016; Laffitte et al., 2016; Espiau et al., 2017; Clayton, 2019). While cells are adapting to the new environment and gradually reaching homeostasis, significant fluctuations in protein and metabolites levels may also result from differences on the rates of differentiation from procyclic to metacyclics that takes place even in axenic conditions (Cysne-Finkelstein et al., 1998; Spath and Beverley, 2001; da Silva et al., 2015), as well as the occurrence of genomic alterations, such as single nucleotide polymorphisms and changes in gene copy numbers (Sinha et al., 2018). Although some pinpoint phenotypic alterations have been associated with attenuation after long-term axenic cultivation, several other concomitant metabolic alterations are likely to occur. In agreement, the previous comparative proteomic analysis of the SIVP promastigotes generated through long-term axenic cultures has revealed decreased virulence, which was validated herein, and several concomitant alterations in protein expression profile (Magalhães et al., 2014).

Changes in promastigotes' cell surface are determinants of host cells interactions, and are frequently associated to decreased promastigotes' infectivity. Metacyclogenesis affects mainly the *Leishmania spp* interactions with surface molecules on the vector's foregut, but also the parasite infectivity to the vertebrate host (da Silva and Sacks, 1987; McConville et al., 1992; Cysne-Finkelstein et al., 1998; Moreira et al., 2012), and correlates with modifications on phosphoglycan surface molecules, especially in LPG (Saraiva et al., 1995; Muskus and Marin Villa, 2002). Nevertheless, the lower infectivity of the *L. amazonensis* SIVP promastigotes could not be correlated to alterations in the percentage of metacyclics: both R0 and R60 displayed low numbers of metacyclics at the stationary growth phase. Therefore, other alterations rather than the metacyclogenesis seem to be important for *L. amazonensis* infectivity. In this sense, it has been shown that deficiency in LPG, as well as other PG containing molecules seems to be not sufficient to impair virulence in *L. mexicana* (Garami et al., 2001), a more closely phylogenetic related species to *L. amazonensis*.

Cellular immune responses are also key determinants in the course of *L. amazonensis* infection in mice. Besides the host inflammatory status, they may also reflect its ability to control parasite multiplication (McMahon-Pratt and Alexander, 2004; Soong et al., 2012; Carneiro et al., 2015; Maspi et al., 2016; Conceicao-Silva et al., 2018). IFN-γ is a biomarker of both inflammatory responses and activation of microbicidal mechanisms. It is important, through the induction of chemokine expression, for migration of CD^+^ T cells and macrophages to lesion site and control of parasite replication (Carneiro et al., 2015). On the other hand, IL-10 is associated with macrophage deactivation and progressive infection (McMahon-Pratt and Alexander, 2004; Soong et al., 2012). Infection with R60 resulted in production of significant increased levels of IFN-γ, but similar levels of IL-10, which correlated with lower parasite burden and decreased lesion size, in comparison to R0. Therefore, the SIVP process induced metabolic changes in R60 that led to significant alterations in inflammatory responses upon infection in BALB/c mice, which correlated to improved control of parasite loads, at early stages of infection.

“Clustering in a heatmap the 66 compounds that were significantly altered in the SIVP metabolome”, a very dymanic and continous adaptation process was disclosed. Altogether fatty acids, phospholipids, organic esthers, and lipids composed the major biochemical category of metabolites that changed during SIVP, suggesting that alterations in their metabolism were critical for the adaptation of *L. amazonensis* to long-term axenic conditions.

Among the identified fatty acids, linoleic acid, arachidic acid, 9-HODE, and palmitic acid showed a tendency of rising up, during SIVP, while stearic, and oleic acids decreased in R10 and R40, reaching similar levels in R60, as compared to R0. Fatty acids metabolism alters significantly the cell membrane fluidity and transport of substances and regulates neutrophil recruitment, host inflammatory responses, affecting several pathways related to trypanosomatid's virulence profile (Uttaro, 2014; Rodrigues et al., 2015). In agreement, these fatty acids seem to be quite important for amastigogenesis and their survival in the vertebrate host. Kloehn et al. (2015) have described that palmitic and stearic acid levels were similar in the host's serum and tissue amastigotes, but lower in cultivated promastigotes. In addition, oleic and linoleic acids were also significantly higher in promastigotes when compared to tissue amastigotes, while levels in amastigotes were higher compared to the host's plasma, and would be then predominantly synthesized by the parasite. Since mammalian hosts do not synthesize linoleic acid, amastigotes are dependent on the *de novo* synthesis of this compound (Kloehn et al., 2015). Therefore, increased levels of linoleic acid in promastigotes may be important for the early stages of amastigote differentiation and multiplication in the vertebrate host. Linoleic acid is produced from oleic acid by the action of desaturases, while oleic acid results from a desaturation of stearic acid, which ends from elongation of palmitic acid (Maldonado et al., 2006; Alloatti and Uttaro, 2011). Thus, the relative abundance of these fatty acids in the metabolomic analysis of SIVP promastigotes suggests that, while palmitic acid is constantly formed from the retooling of carbohydrates and protein metabolism, oleic and stearic acids are consumed to generate linoleic acid and its metabolites.

Arachidonic acid (AA) can modulate the expression of cyclooxygenase-2 (COX-2), a pivotal enzyme involved in skin inflammation and tissue reparation, which is responsible for the synthesis of prostaglandins (Chene et al., 2007). Various metabolites around AA metabolism were altered alongside the SIVP process, including, linoleic acid, a direct AA precursor and some AA derivates, such as 9-HODE and leukotriene B4. Previous studies have shown that AA induces parasites to release prostaglandins (Reiner and Malemud, 1985; Araujo-Santos et al., 2014), which affect the formation of lipid bodies. Increased levels of AA have been observed in lipid bodies of metacyclic forms and, during *L. chagasi* infection, the inhibition of prostaglandin receptor impairs parasite survival in macrophages (Araujo-Santos et al., 2014). Therefore, parasite-derived prostaglandins play a critical role in macrophage infection and are implicated in virulence. The fact that AA has not been found in the metabolomic fingerprint of SIVP promastigotes suggests a high consumption, given rise to the resultant metabolites of the lipoxygenase and prostaglandin synthase action, such as Leukotriene B4 (LTB4), or that its metabolism is significantly deviated to other pathways, such elongation of stearic acid and synthesis of 9-HODE. LTB4, which can be generated by the action of lipoxygenase on linoleic acid, is known as one of the most potent chemoattractant and activator of leukocytes involved in inflammatory diseases (Yokomizo et al., 2001). Moreover, macrophages from C3H/HePAS resistant mice produced higher levels of LTB4 upon *L. amazonensis* challenge than did those from susceptible BALB/c mice (Serezani et al., 2006). Treatment of human neutrophils with exogenous LTB4, prior to infection, significantly reduced the number of *L. amazonensis* viable parasites (Tavares et al., 2014), suggesting that, regardless of an endogenous or exogenous source, LTB4 might be required for protective responses against *L. amazonensis* infection. The 9 + 13 HODE byproducts of AA are also involved in inflammatory responses through regulation of cell adhesion molecules, neutrophil chemotaxis and degranulation, macrophage superoxide production, PPAR-γ activation, and inhibition of protein kinase C (Vangaveti et al., 2010; Rolin et al., 2014). Thus, 9 + 13 HODE may also assist in the control of neutrophil function, perhaps mitigating excessive neutrophil ROS, degranulation, and cell damage.

The ubiquitous ABC transporters protein family is composed by trans-membrane proteins, which are associated to lipid translocation across the membrane and are quite promiscuous regarding their lipid substrates. ABC transporters were already associated to increased intracellular levels of aPS, cholesterol, ergosterol, palmitic, myristic, linoleic acid, and arachidic acid, leukotriene B4, and other lipids, which were identified by the metabolomics of SIVP (Borst et al., 2000; van Meer et al., 2006; Zhang and Beverley, 2010). Interestingly, significant alterations in *L. donovani* ABC transporter genes have been seen by comparing the genome of early and late passages parasites, that have been associated to decreased virulence, including the presence of single nucleotide polymorphisms (Sinha et al., 2018). In *L. amazonensis*, a potential role of these proteins in cholesterol accumulation can be suggested, since this species lacks a *de novo* mechanism for cholesterol synthesis and, therefore, must scavenge this lipid from the host environment. In order to compensate for the lack of cholesterol synthesis, a lipid importing machinery would be required (De Cicco et al., 2012).

Phosphatidylserine, phenylethanolamine, lyso-phosphatidyletanolamine and phosphatidylinositol, as well as changes at hexadecasphinganine and O-phosphocholine were also detected in the long-term cultivation parasites. These metabolites are destined to the cell membrane and, therefore, also require transportation to their final location (Woehlecke et al., 2003; Wanderley et al., 2006; De Cicco et al., 2012; Campos-Salinas et al., 2013), which can be performed by members of the ABC transporters family. Since the enrichment analysis of metabolomic data revealed statistical significance for the ABC transporter pathway, we performed a qPCR analysis of some ABC transporters genes described in *L. amazonensis*. As expected, the SIVP promastigotes showed significant increase in transcripts for ABC-C, ABC-G2 and ABC-G4 transporters. Recently, the ABC-G2 gene was shown to affect PS exposure and the ABC-G2 *L. major* knockout parasites displaying low PS exposure, are less virulent (Campos-Salinas et al., 2013). On the other hand, we have shown that ABC-G2 expression was increased during SIVP, while parasites accumulate aPS in the cell cytoplasm and expose less in the cell membrane, according to the FACS analysis. This apparent contrast with the previous effect reported for *Leishmania* ABC-G2 may be possibly explained by a competition for the ABC-G2 transporters among PS, other phospholipids, lipids, fatty acids and thiols, which are all increased in *L. amazonensis* SIVP parasites, as previously mentioned. The occurrence of point mutations or pseudogenes in ABC transporter genes, affecting their functionality, cannot be discarded as well, as this alterations have been previously reported in parasites submitted to SIVP (Sinha et al., 2018). Nonetheless the origin of these alterations, the transient non-exposure of aPS may have influenced the SIVP parasite's survival inside the macrophage, and thus, their attenuated profile, since PS exposure has been associated to increased virulence in *Leishmania* (Wanderley et al., 2006; Franca-Costa et al., 2012; Rochael et al., 2013).

In addition to ABC transporters, significant modulation on the expression of proteins related to membrane vesicular trafficking and remodeling, such as small GTP-binding protein Rab1, protein transport Sec13, actin and tubulin as well as of membrane-associated proteins, including the small myristoylated protein 3, enolase, and glucose regulated protein of 78 kDa were associated to SIVP, as disclosed by proteomics (Magalhães et al., 2014). The metabolite UDPG is essential for the N-glycosylation of membrane proteins and is linked to the metabolic pathway that includes UMP (uridine monophosphate), glycerol- 1 phosphate (Glc-1-P), ureidopropionate (Silva Pereira and Jackson, 2018); displaying decreased abundance in R60. In agreement, the expression of UDP-Glucose pyrophosphorilase, an enzyme that produces UDPG (Uridine Diphosphate-Glucose) and pyrophosphate (PPi), from Glc-1-P and UTP was also altered in SIVP proteomics (Magalhães et al., 2014). Myristoylation of proteins involves the addition of a myristoyl group, derived from myristic acid, and allows for weak protein–protein and protein–lipid interactions and, thus, links protein and lipids metabolism. Protein N-linked glycosylation is also an important mechanism for folding and/or oligomerization, and consequently to the correct trafficking and cellular addressing of proteins. Inhibition of myristoylation has been shown to have pleiotropic effects in *Leishmania* (Wright et al., 2015). Small myristoylated proteins localize in the inner leaflet of the *Leishmania* flagellar membrane and are associated with vesicular trafficking, stabilization of the flagellar membrane sterol- and sphingolipds reach domains (Tull et al., 2010; Wright et al., 2015). These functions are, therefore, consistent with the alterations in transcripts levels of the small myristoyled protein gene, as well as in the proteome (Magalhães et al., 2014) and metabolome of the SVIP promastigotes, especially during the first 10 SIVP.

Fatty acid metabolism is significantly affected during *Leishmania* lifecycle (Bouazizi-Ben Messaoud et al., 2017), reflecting the availability of nutrients and precursors that parasites found in a given environmental and the ability of the parasite to synthesize, metabolize or use them to compose membrane structures. Amastigotes obtain fatty acids from complex host lipids and use them as an alternative carbon source, but the lack of glyoxylate cycle enzymes needed to use acetyl-CoA in gluconeogenesis, suggests that *Leishmania* is unable to use fatty acids as the sole carbon source. While non-dividing promastigotes also use fatty acid β-oxidation as a secondary carbon source, this process occurs to a negligible extent in actively dividing promastigotes (McConville and Naderer, 2011). Accordingly, parasites recently recovered from the restricted nutritional microenvironment of host cells, are still metabolically adapted for the intracellular lifestyle, characterized by reduced metabolic activity (Burchmore and Barrett, 2001; Alcolea et al., 2010; Jara et al., 2017), decreased glucose utilization and increased fatty acid β-oxidation (Saunders et al., 2014). In contrast, in axenic culture, a less hostile environment than the parasitophorous vacuole and plentiful in nutrients, promastigotes channeled cell metabolism to anabolic reactions to support replication. The central metabolic hub of this adaptive metabolic process during SIVP seems to be the fatty acids biosynthesis, which requires adequate concentration of NADPH and acetyl-CoA. NADPH is generated in the pentose phosphate pathway (PPP), by the malic enzyme, which oxidizes malate into pyruvate and CO_2_. Consistently, significant alterations in succinate, glycerol 1-phosphate and acetol were detected in the SIVP metabolomics. Similarly, cancer cells overexpress the malic enzyme to keep with the high demand of fatty acids (Sarfraz et al., 2018). Isocitrate dehydrogenase is another important enzyme that enables aerobic cells to fulfill their requirement for NADPH (Sarfraz et al., 2018). Likewise, changes in the levels of malic enzyme, isocitrate dehydrogenase and 2-hydroxy-3-oxopropionate reductase (another enzyme involved in the production of NADPH) that have been reported in promastigotes of *L. infantum* (Louassini et al., 1999) were also observed in the proteomic analysis of *L. amazonensis* promastigotes after 30 SIVP (Magalhães et al., 2014) and in the qPCR analysis of their transcripts in R0, R10, R40, and R60.

The increased intracellular levels of fatty acids observed in the metabolomic analyses may also reflect this metabolic retooling, which activates PPP to supply the cell with NADPH and nitrogenous bases for DNA and RNA synthesis, in order to support growth rates of SIVP promastigotes. To confirm this hypothesis, the mRNA levels of cyclin, a protein involved with cell cycle progression (Ali et al., 2010) and previously shown increased in the proteomics of SIVP promastigotes, was also measured by qPCR. As expected, cyclin expression increased significantly in R10 when compared to R0, reaching afterwards, in R40 and R60, similar levels to R0.

The shift to anabolic reactions requires intracellular reducing power of NADPH and a high activity of the parasite's redox system (Ilari et al., 2017). In agreement, the metabolome disclosed changes on the levels of proline, ornithine, citruline, spermidine, and putrescine, which are linked to parasite's redox system. Interestingly, pipecolate, another metabolite related to proline-ornithine metabolism, but not previously reported in *Leishmania*, has been involved in protection of mammalian cells against oxidative stress, also increased during SIVP. The involvement of this pathway was corroborated by increased mRNA levels of trypanothione reductase and mitochondrial tryparedoxin peroxidase, as detected by qPCR in long-term cultivated parasites and in proteomics (Magalhães et al., 2014), inferring an overload of the glutathione pathway. S-adenosylhomocysteine was found increased in our analysis and could be linked to both glutathione and glycerophospholipid metabolisms (Kennedy pathway) (Pessi et al., 2005). In accordance to our main hypothesis, spermidine was synthesized from the pool of putrescine, while phosphatidylethanolamine originated from the glycerophospholipid metabolism. Therefore, it is possible that the SAM pathway had changed, leading to the increased levels of S-adenosylhomocysteine and betaine, observed during SIVP. Consistently, regulation of S-adenosylmethionine synthetase was associated to SIVP (Magalhães et al., 2014), further supporting this hypothesis.

Besides its significant role to control the parasite's intracellular redox state, trypanothione reductase is important in drug resistance in *Leishmania* and is inhibited by Sb^III^ in a NADPH-dependent reaction (Cunningham and Fairlamb, 1995; Baiocco et al., 2009; Cole, 2014). Moreover, ABC transporters are also involved in efflux of thiol and GSH-conjugated compounds, or may in same cases be stimulated by GSH (Leprohon et al., 2006). Accordingly, various ABC transporters subfamilies have been identified in *Leishmania*, which are responsible for the cellular transport of several compounds and have been connected with resistance to drugs, including antimonial compounds (Leprohon et al., 2006; Sauvage et al., 2009). Thus, it is plausible that long-term cultivated parasites may be more sensitive to drugs targeting trypanothione reductase and produce more reactive oxygen species (ROS). We hypothesized that fatty acid metabolism alterations induced glutathione system disequilibrium, with increased production of ROS that compromised the detox process and turned the parasites more sensitive to antimonials. In agreement, when we used Sb^III^ to disturb the redox system, SIVP parasites were more sensitive when compared to R0, again suggesting that the redox system was activated to counter balanced the oxidative state caused by anabolic reactions. Interestingly, the R60 parasites produced significantly higher levels of ROS than the R0 reference parasites, explaining, at least in part, the increased sensibility of R60 parasites to antimony. These results are also in agreement with significant alterations in glutathione metabolism and ABC transporters pathways, as indicated by qPCR results.

The SIVP process was accompanied by significant fluctuations in levels of the identified metabolites, indicating that *Leishmania* adaptation to a new environmental condition is a very dynamic process. As previously pointed out, successive changes in predominance of clones with different proliferative capacity and metabolic fitness may globally interfere with metabolite's levels. In addition, significant heterogeneity may result from developmental differentiation from procyclic to metacyclics and from genome alterations that takes place even in axenic conditions (Sinha et al., 2018). In agreement, late passages *L. donovani* parasites have significant higher numbers of pseudogenes, single nucleotide polymorphisms and alterations in gene copy number, as compared to early passages parasites, which directly impact in RNA and protein levels and activity (Sinha et al., 2018). Moreover, at each metabolomic measure, the individual metabolites levels may reflect reduced or increased production, consumption or their absorption from environment or host. For instance, it has been shown that, when the uptake rates were corrected for diffusion, the purine bases adenine and hypoxanthine were transported at a significantly slower rate than the purine nucleosides adenosine and inosine. In addition, adenine and hypoxanthine inhibited the uptake of one another competitively (Hansen et al., 1982). Considering these metabolites, it is also known that kinetoplastid protozoa are auxotrophs for purine bases, since *de novo* biosynthetic pathway is completely absent (Ullman and Carter, 1997). They are therefore dependent on recycling pre-formed purine nucleotides and acquiring purines from the host/environment. The salvage pathway recovers purines (adenine and guanine) from the degradation products of nucleotide metabolism and from hypoxanthine and xanthine. Then, we may hypothesize that levels of components of the nucleotide metabolism may decrease from R0 and R10, while cells are adapting from a quiescent condition (amastigotes in tissues), with low intracellular stocks, when DNA replication and energy metabolism is down-regulated to a highly replicative condition in an environment plenty of nutrients. Later, when cells are tending to achieve homeostasis, levels may increase, as gradually seen in R40 and R60. In agreement, proteome comparisons between purine-starved and purine-replete parasites have revealed a temporal and coordinated response to purine starvation (Martin et al., 2014). Purine transporters and enzymes involved in acquisition at the cell surface are up regulated within a few hours of purine removal from the media, while other key purine salvage components are up regulated later in the time-course and more modestly. Interestingly, significant fluctuations in protein expression could also be seen even in a short period of time (48 h), in deprived cells, unveiling extensive reprogramming of the parasite proteome and metabolome to adapt to the stress condition (Martin et al., 2014).

Some of the identified compounds were not or are rarely described in *Leishmania*, including the 2,6-diaminopimelic acid, a carbohydrate found in bacteria cell wall. According to Opperdoes et al. (2016), among trypanosomatids, *Leptomonas* and *Crithidia*, members of the Leishmaniinae subfamily, have acquired the capacity to convert this compound to lysine. Therefore, it is conceivable that *Leishmania* may also be able to metabolize this compound. Another compound is 2,6-farnesol, an isoprenoid resulting from mevalonic acid pathway, linked to the synthesis of sterols and dolichol. According to these same authors, enzymes synthesizing this long chain isoprenoids have been scarcely studied in trypanosomatids. The findings of these rarely seen compounds support the potential of our fingerprinting metabolomics approach for better uncover of *Leishmania* metabolism.

It is noteworthy that plausible connections could be established among our findings and the previous preoteomic profile reported for the SIVP process (Magalhães et al., 2014). Therefore, we have merged proteome and metabolome data sets for a holistic view of metabolic pathways changed during SIVP process. Consistent with the predominance of lipids identified by metabolomics, the biochemical model led to a central hypothesis based on the adaptation to a nutrient rich environment. Promastigote's metabolism of carbohydrates (glicolitic and gluconeogenesis, pentose-phosphate pathway, acetol degradation), amino acid (amino acid biosynthesis and degradation, β-alanine, glycine and serine and arginase metabolisms) and purine, pyrimidine metabolism were retooled to supply the demands from increases in energy-intensive processes such as genome replication and protein synthesis and lipid metabolism (glycerophospholipids, fatty-acids biosynthesis), altering the cellular oxidative state. As a consequence, the redox homeostasis processes (SAM and glutathione metabolisms) changed to control the intracellular oxidative stress, resultant from the intense anabolic metabolism. A concerted alteration in expression of proteins associated with intracellular and membrane traffic of lipids, such as ABC transporters, led probably to significant remodeling of membrane lipid composition. This promastigotes' cell membrane remodeling and the lipid mediators may have, finally, affected significantly the parasite induced host inflammatory responses and the control of parasite replication, during early stages of infection.

In summary, our biochemical model point out to a very dynamic and continuous metabolic reprogramming process and the high capacity of *L. amazonensis* to adapt to the nutritional availability, modulating its metabolism and phenotype. This process was accompanied by changes in membrane remodeling, which, in turn, shaped parasite-host cells interactions to an attenuated profile. The clonal propagation structure, the plasticity in genome and gene expression are key elements to maintain heterogeneity inside each population, as well as to understand the alterations seen in metabolites' levels during the SIVP process. Although changes were detected in various metabolic pathways, most of them seemed to be metabolic adaptations to central alterations in the fatty acid and carbon metabolism, which acts as a metabolic hub, driving NADPH production. Anabolic reactions generate an oxidized intracellular state that is counterbalanced by the reducing power of NADPH and the parasite's redox system. Alterations in fatty acid composition secondarily affected parasite virulence due to membrane remodeling with an overall impact in chemotaxis and host inflammatory responses, at the early stages of *L. amazonensis* infection.

## Data Availability Statement

The raw data supporting the conclusions of this manuscript will be made available by the authors, without undue reservation, to any qualified researcher.

## Ethics Statement

Experiments were performed in compliance with the National Guidelines of the Institutional Animal Care and Use Committee for the Ethical Handling of Research Animals (CEUA) from the Federal University of Minas Gerais (UFMG) (Law number 11.794, 2008), which approved under protocol CETEA number 240/2016.

## Author Contributions

FC, JT, AF, GM, and CB conceived and designed the experiments. FC, JT, AS, AC, LF, SM, AA, LO, DM, AD, and TR performed the experiments. ÁL-G, FC, SM, JT, CB, GM, and AF analyzed the data. JT, FC, ÁL-G, DB, and AF wrote the paper.

### Conflict of Interest

The authors declare that the research was conducted in the absence of any commercial or financial relationships that could be construed as a potential conflict of interest.

## Supplementary Material

Supplemental Figure 1 Growth curves for R0, R10, R40, and R60 promastigotes, cultivated in medium M199 pH7.4, 26°C. Results correspond to average and SD of three different countings; two different experiments were performed.

Supplemental Figure 2 Promastigotes from R0 and R60 were cultivated in M199 medium supplemented with 10% BFS, at 26°C, collected at 4th and 7th days of growth, separated by ficoll gradient and submitted to flow citometrry (BD, LSRFortessa cell analyser) to determine the gates for procyclic **(A)** and metacyclic **(B)** populations. To differentiate these populations, a merged graph **(C)** was constructed, showing the procyclic population in blue (circle) and the metacyclic population in red (square).

Supplemental Figure 3 Scores plot for PCA-X. Models built with the filtered data set for each analytical technique obtained from R0, R10, R40, and R60 extract samples, provided by SINCA p+ statistical software demonstrating evident QC overlap (black) and good separation among the groups in unsupervised analysis. The predictive coefficients values demonstrated a strong statistical trend regardless the analytical techniques, in CE-MS [R^2^ = 0.904, Q^2^ = 0.641], GC-MS [R^2^ = 0.715, Q^2^ = 0.42] and LC-MS [R^2^ = 0.314, Q^2^ = 0.152, in negative mode and R^2^ = 0.773, Q^2^ = 0.193 in positive mode].

Supplemental Figure 4 Scores plot for PLS-DA. Models built with the filtered data set for each analytical technique obtained from R0, R10, R40, and R60 extract samples, provided by SINCA p+ statistical software demonstrating evident separation among the groups in supervised analysis, except for the LC-MS positive mode. The predictive coefficients values demonstrated a strong statistical trend regardless the analytical techniques, for CE-MS [R^2^ = 0.983, Q^2^ = 0.947], GC-MS [R^2^ = 0.594, Q^2^ = 0.342] and LC-MS [R^2^ = 0.582, Q^2^ = 0.262 in positive, and R^2^ = 0.744, Q^2^ = 0.201, in negative mode].

Supplemental Figure 5 Determination of gates for aPS exposure. **(A)** Unlabeled parasites. **(B)** Parasites marked only with PE annexinV (aPS exposure). **(C)** Parasites marked only with 7-AAD (unviable parasites).

Supplemental Table 1 Primers for RT-PCR of selected loci.

Supplemental Table 2 List of all metabolites identified by the metabolomic multiplatform fingerprint approach. Confirmed indicates that the compound was putative or truly identified; Feature correspond to a peak or signal that represents a potential metabolite, which should be further identified, according to mass, charge and retention time. M/Z: molecular ion described by its molecular weight in Daltons; RT: retention time for each feature; AVG represent the average of the relative ion abundance present in each investigated group and each metabolite; pV: calculated *p*-value; Compound ID: compound's identification number in its respective database; Mass Theor: theoretical molecular mass; MH formula: theoretical formula; Score: probability of matched score from each identified metabolite; Ppm error: ppm error between the compound molecular weight and the found compound molecular weight.
