# Supplementary figures and images for: Mapping Alterations Induced by Long-Term Axenic Cultivation of Leishmania amazonensis Promastigotes With a Multiplatform Metabolomic Fingerprint Approach
Source: Front Cell Infect Microbiol. 2019 Dec 4;9:403. doi: 10.3389/fcimb.2019.00403 (PMC6904349; doi:10.3389/fcimb.2019.00403)

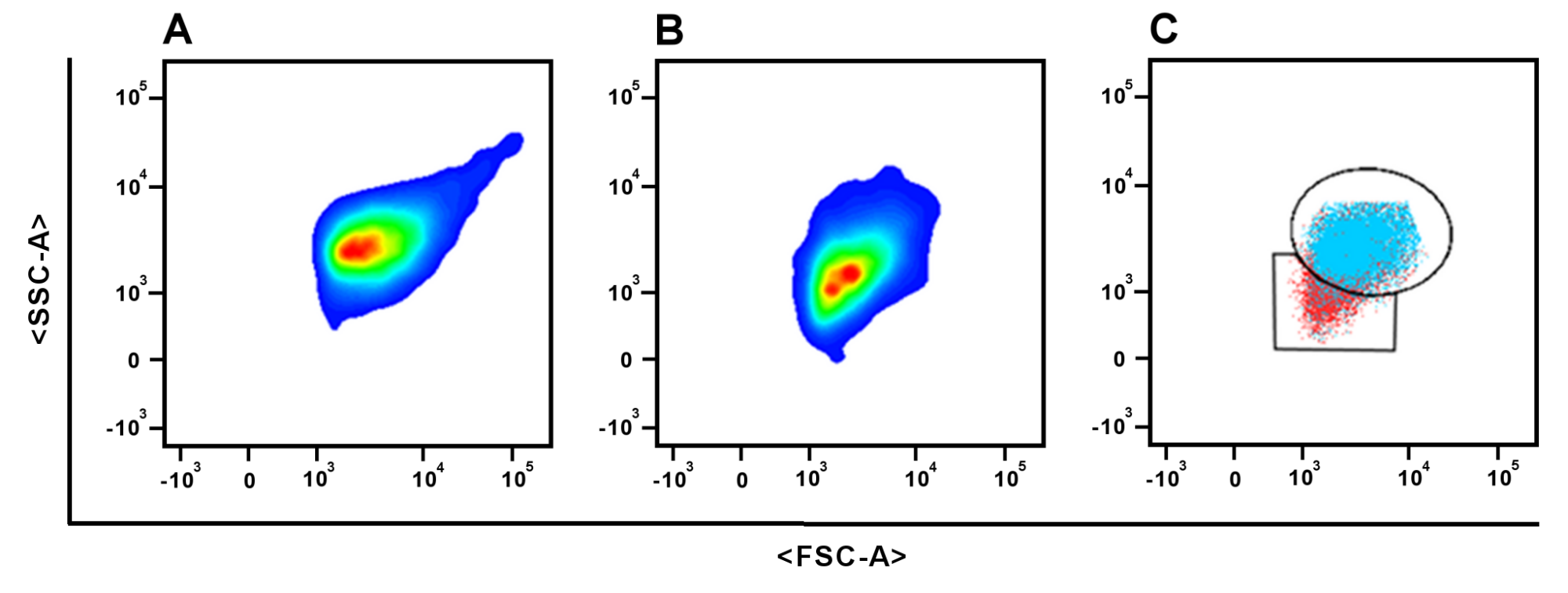

Supplement: Supplemental Figure 2 — Promastigotes from R0 and R60 were cultivated in M199 medium supplemented with 10% BFS, at 26°C, collected at 4th and 7th days of growth, separated by ficoll gradient and submitted to flow citometrry (BD, LSRFortessa cell analyser) to determine the gates for procyclic (A) and metacyclic (B) populations. To differentiate these populations, a merged graph (C) was constructed, showing the procyclic population in blue (circle) and the metacyclic population in red (square). [file Image_2.tif]

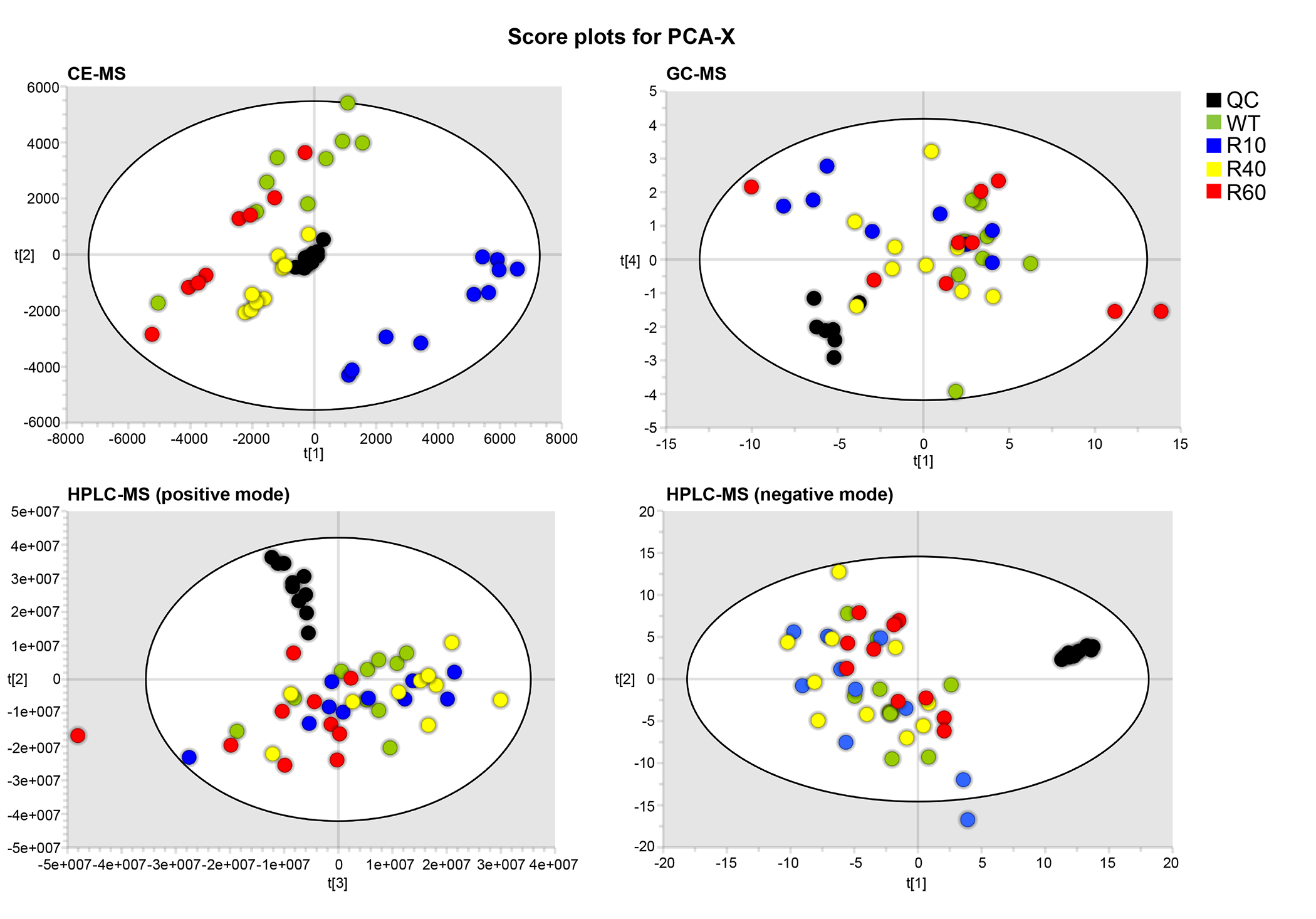

Supplement: Supplemental Figure 3 — Scores plot for PCA-X. Models built with the filtered data set for each analytical technique obtained from R0, R10, R40, and R60 extract samples, provided by SINCA p+ statistical software demonstrating evident QC overlap (black) and good separation among the groups in unsupervised analysis. The predictive coefficients values demonstrated a strong statistical trend regardless the analytical techniques, in CE-MS [R2 = 0.904, Q2 = 0.641], GC-MS [R2 = 0.715, Q2 = 0.42] and LC-MS [R2 = 0.314, Q2 = 0.152, in negative mode and R2 = 0.773, Q2 = 0.193 in positive mode]. [file Image_3.TIF]

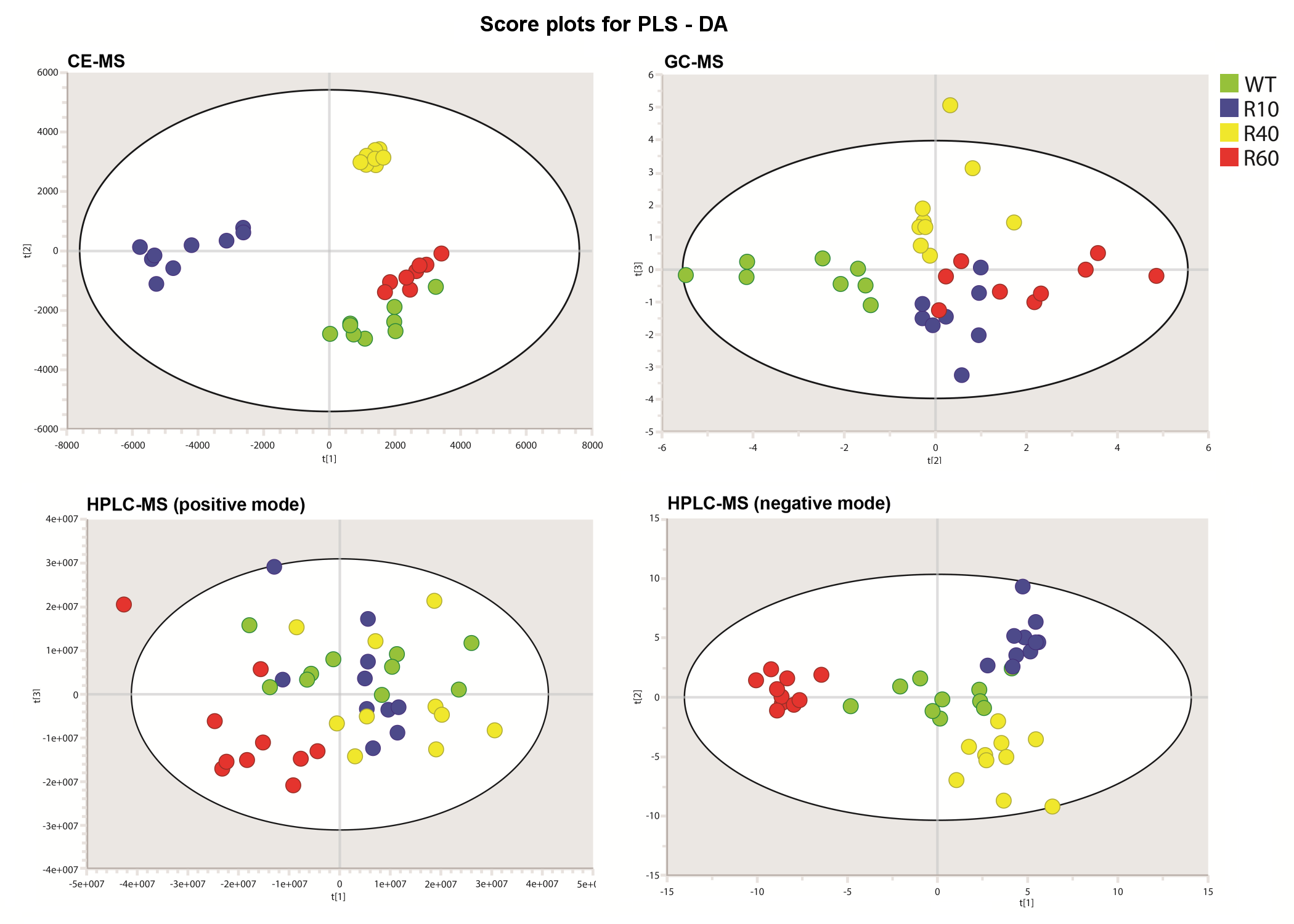

Supplement: Supplemental Figure 4 — Scores plot for PLS-DA. Models built with the filtered data set for each analytical technique obtained from R0, R10, R40, and R60 extract samples, provided by SINCA p+ statistical software demonstrating evident separation among the groups in supervised analysis, except for the LC-MS positive mode. The predictive coefficients values demonstrated a strong statistical trend regardless the analytical techniques, for CE-MS [R2 = 0.983, Q2 = 0.947], GC-MS [R2 = 0.594, Q2 = 0.342] and LC-MS [R2 = 0.582, Q2 = 0.262 in positive, and R2 = 0.744, Q2 = 0.201, in negative mode]. [file Image_4.TIF]

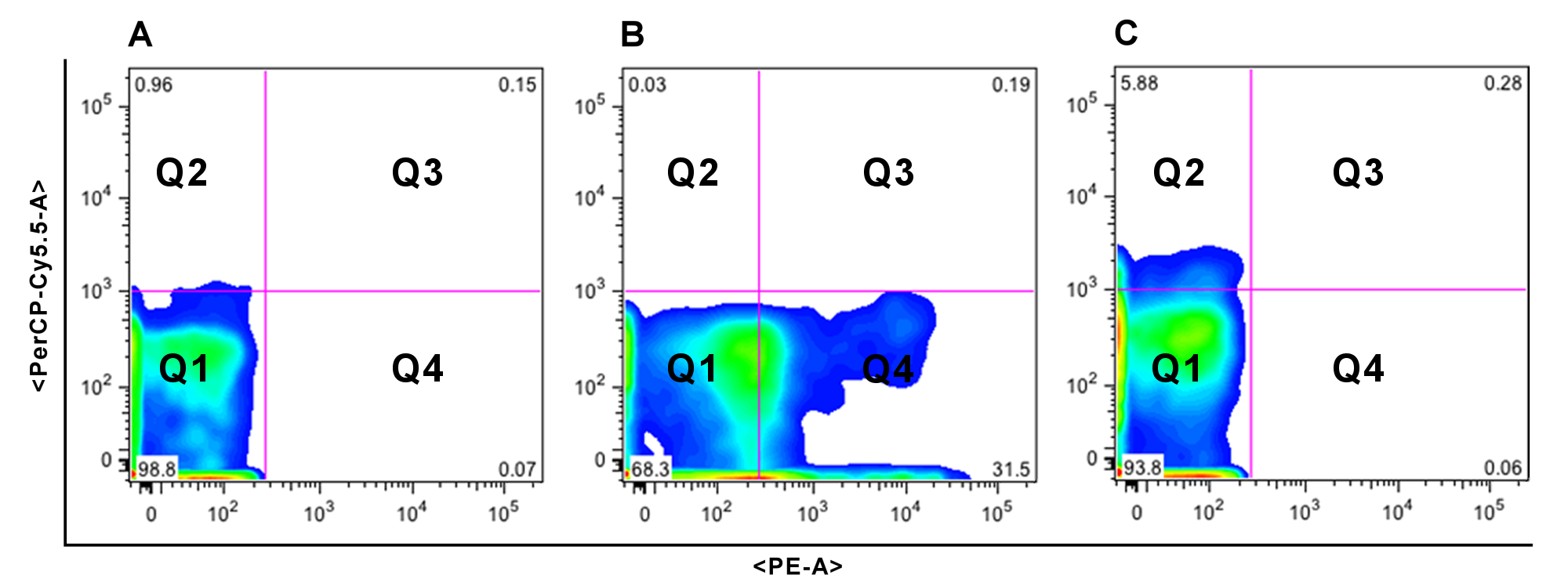

Supplement: Supplemental Figure 5 — Determination of gates for aPS exposure. (A) Unlabeled parasites. (B) Parasites marked only with PE annexinV (aPS exposure). (C) Parasites marked only with 7-AAD (unviable parasites). [file Image_5.TIF]
