# Supplementary material for: Mapping Alterations Induced by Long-Term Axenic Cultivation of Leishmania amazonensis Promastigotes With a Multiplatform Metabolomic Fingerprint Approach
Source: Front Cell Infect Microbiol. 2019 Dec 4;9:403. doi: 10.3389/fcimb.2019.00403 (PMC6904349; doi:10.3389/fcimb.2019.00403)
Supplement: Supplemental Table 1 — Primers for RT-PCR of selected loci. [file Table_1.pdf]

Table 1 - Primers for RT-PCR of selected loci

Protein name		PRIMERS	
GAPDH		R	CACTTGTCGTGAATGGAAAG
		F	AGTTGACTCCACCACCACTT
**Cluster 1**			
**1**	N-acetylglucosamine 1 phosphate	R	TGAACGACGAGGAGCACAAG
		F	TCCTCTACCTCTTCGGCGAT
**2**	Cyclin	R	ACGAACCCAAAAACACAGGC
		F	GCACCACACTTGAACTCCATTC
**3**	Isocitrate dehydrogenase	R	TTGATGGTGCTGTTGTCCAC
		F	AACAGCGACCTCGTCAAGTT
**4**	Small myristoylated protein	R	ATCACAGACCACCCGTTCTC
		F	TCTTGCTGTCCTTGCCGAAC
**5**	Cytosolic tryparedoxin peroxidase	R	TGATGGTCTTGCCGTTGATA
		F	TTTCAGTTCGTGGAGAAGCA
**6**	Malic enzyme	R	CCGTCGTCTCGTAGTGTTCA
		F	CGGAGGTTATGCCAGATGAT
**7**	Cyclopropane-fatty-acyl-phospholipid syntahse	R	CAGTAAGACGACCATGAGCA
		F	GAAGCCAAAGTTATGCAGGT
**8**	ABC transporter subfamily G member 4	R	CTACTTCCTCGCCAAGAACA
		F	GGTGAAGGTGGATCATGAAA
**Cluster 2**			
**9**	2-hydroxy-3-oxopropionate reductase	R	CCGAAGACGTAGGCGACAAA
		F	TGACGTGATGGAGATCGTGT
**10**	Mitochondrial tryparedoxin peroxidase	R	GCTGAAGTACCCCTCGACAG
		F	GATCATTGCGTTTTCGGATCG
**Cluster 3**			
**11**	Trypanothione reductase	R	TACCAACGTAGGCGACACTC
		F	CGAAGGACCACGAAACCG
**12**	ABC transporter subfamily C	R	CGTCGCGATAGTTTTCATCT
		F	TCGGGATCATAAAGAACGAC
**13**	ABC transporter subfamily G member 2	R	TCGTACTTCGACGACGTGAT
		F	ACCCGATGGACTCGAAGTAG
