# Supplementary material for: Mapping Alterations Induced by Long-Term Axenic Cultivation of Leishmania amazonensis Promastigotes With a Multiplatform Metabolomic Fingerprint Approach
Source: Front Cell Infect Microbiol. 2019 Dec 4;9:403. doi: 10.3389/fcimb.2019.00403 (PMC6904349; doi:10.3389/fcimb.2019.00403)
Supplement: Supplemental Table 2 — List of all metabolites identified by the metabolomic multiplatform fingerprint approach. Confirmed indicates that the compound was putative or truly identified; Feature correspond to a peak or signal that represents a potential metabolite, which should be further identified, according to mass, charge and retention time. M/Z: molecular ion described by its molecular weight in Daltons; RT: retention time for each feature; AVG represent the average of the relative ion abundance present in each investigated group and each metabolite; pV: calculated p-value; Compound ID: compound's identification number in its respective database; Mass Theor: theoretical molecular mass; MH formula: theoretical formula; Score: probability of matched score from each identified metabolite; Ppm error: ppm error between the compound molecular weight and the found compound molecular weight. [file Table_2.pdf]

Compound	Confirmed?	Technique	Feature	M/Z	RT	AVG R0	AVG Log2 R0	AVG R10	AVG Log2 R10	AVG R40	AVG Log2 R40	AVG R60	AVG Log2 R60	FC L 2 R0xR10	FC L 2 R0xR40	FC L 2 R0xR60	pV R0xR10	pV R0xR40	pV R0xR60	COMPOUND ID	Mass Theor	Formula MH	Score MH	Error (PPM)
Betaine	Y	CEMS	117.0791@14.30723	117,0791	14,31	8994034,11	23,10	11051796,30	23,40	8694139,89	23,05	8589758,63	23,03	0,30	-0,05	-0,07	4,11E-04	3,49E-01	3,39E-01	C00719	117,0790	C5H11NO2	47,61	0,83
Citrulline	Y	CEMS	175.0962@15.425029	175,0962	15,43	62073,56	15,92	82071,60	16,32	65902,22	16,01	37993,29	15,21	0,40	0,09	-0,71	1,72E-02	1,90E-01	6,99E-04	C00327	175,0957	C6H13N3O3	44,57	2,92
Histidine	Y	CEMS	155.0695@11.164937	155,0695	11,16	819533,33	19,64	818775,30	19,64	644291,56	19,30	667473,50	19,35	0,00	-0,35	-0,30	7,80E-01	7,82E-04	2,06E-02	C00768	155,0695	C6H9N3O2	46,14	-0,02
Ornithine	Y	CEMS	132.0898@10.587783	132,0898	10,59	296857,22	18,18	399255,40	18,61	179980,78	17,46	176306,38	17,43	0,43	-0,72	-0,75	1,48E-06	4,09E-09	9,29E-08	C00515	132,0899	C5H12N2O2	44,67	-0,56
Phenylalanine	Y	CEMS	165.0795@15.426141	165,0795	15,43	1579780,11	20,59	1824158,70	20,80	1464672,33	20,48	1180895,88	20,17	0,21	-0,11	-0,42	6,49E-02	2,76E-01	9,50E-03	C02265	165,0790	C9H11NO2	87,43	3,06
Pipecolate	Y	CEMS	129.079@14.611996	129,079	14,61	13159196,89	23,65	21570584,20	24,36	16288555,22	23,96	12338079,63	23,56	0,71	0,31	-0,09	1,01E-09	8,72E-05	1,42E-01	C00408	129,0790	C6H11NO2	35,78	0,16
Putrescine	Y	CEMS	88.1005@7.566636	88,1005	7,57	913048,00	19,80	970066,10	19,89	802026,22	19,61	647558,71	19,30	0,09	-0,19	-0,50	2,64E-01	2,21E-02	7,06E-05	C00134	88,1000	C4H12N2	46,69	5,69
S-Adenosylhomocysteine	Y	CEMS	384.1226@12.909194	384,1226	12,91	57290,44	15,81	56994,70	15,80	74049,44	16,18	52867,75	15,69	-0,01	0,37	-0,12	9,42E-01	1,11E-04	3,55E-01	C00021	384,1216	C14H20N6O5S	45,56	2,57
Spermidine	Y	CEMS	145.1578@7.340344	145,1578	7,34	631872,56	19,27	314954,80	18,26	250369,56	17,93	326455,50	18,32	-1,00	-1,34	-0,95	1,68E-02	1,65E-04	2,74E-02	C00315	145,1579	C7H19N3	41,62	-0,67
Triethanolamine	Y	CEMS	149.1053@14.514858	149,1053	14,51	173636,22	17,41	161997,70	17,31	177033,78	17,43	148606,00	17,18	-0,10	0,03	-0,22	4,34E-01	8,04E-01	1,10E-01	C06771	149,1052	C6H15NO3	66,68	0,71
3-Ureidopropionate	Putative	CEMS	132.0535@14.809702	132,0535	14,81	581005,67	19,15	414268,00	18,66	406619,33	18,63	435194,63	18,73	-0,49	-0,51	-0,42	2,09E-04	6,13E-05	3,70E-03	C02642	132,0535	C4H8N2O3	64,94	0,02
Ammonium lactate	Putative	CEMS	107.0584@13.207008	107,0584	13,21	706024,11	19,43	503673,80	18,94	566976,22	19,11	571537,63	19,12	-0,49	-0,32	-0,30	2,05E-03	2,55E-02	6,37E-02	D02920	107,0582	C3H9NO3	40,88	1,47
Glu-Arg	Putative	CEMS	303.1548@12.787672	303,1548	12,79	22742,75	14,47	17499,80	14,10	31308,89	14,93	36678,25	15,16	-0,38	0,46	0,69	4,21E-03	1,84E-04	2,50E-06	23759	303,1543	C11H21N5O5	40,72	1,76
Gly-Leu-Lys	Putative	CEMS	316.2099@11.474592	316,2099	11,47	46295,13	15,50	47030,14	15,52	42405,14	15,37	36076,67	15,14	0,02	-0,13	-0,36	1,21E-01	1,52E-01	1,42E-01	16534	316,2111	C14H28N4O4	38,53	-3,67
Isoglutamine	Putative	CEMS	146.0691@15.13938	146,0691	15,14	1979786,11	20,92	1137626,80	20,12	1059648,20	20,02	1888117,71	20,85	-0,80	-0,90	-0,07	4,22E-05	1,77E-05	5,15E-01	C16673	146,0691	C5H10N2O3	35,62	-0,26
Lysine	Putative	CEMS	146.1056@10.652979	146,1056	10,65	2211967,89	21,08	2257389,60	21,11	3121917,33	21,57	2520913,50	21,27	0,03	0,50	0,19	7,14E-01	2,37E-07	5,77E-02	C00047	146,1055	C6H14N2O2	35,05	0,48
N-Sulfonyloxy-PhIP	Putative	CEMS	320.061@12.946461	320,061	12,95	23017,00	14,49	32758,10	15,00	23073,43	14,49	17407,00	14,09	0,51	0,00	-0,40	2,54E-01	9,83E-01	3,29E-02	C20288	320,0579	C13H12N4O4S	43,12	9,71
O-Acetylserine	Putative	CEMS	147.0534@15.303778	147,0534	15,30	1793154,00	20,77	1554672,40	20,57	1171379,00	20,16	1442723,63	20,46	-0,21	-0,61	-0,31	6,76E-02	1,85E-03	1,26E-02	C00979	147,0532	C5H9NO4	37,47	1,62
γ-D-Glutamylglycine	Putative	CEMS	204.0747@16.411814	204,0747	16,41	711246,89	19,44	949599,30	19,86	634870,00	19,28	662604,63	19,34	0,42	-0,16	-0,10	7,77E-06	3,29E-02	1,53E-01	24059	204,0746	C7H12N2O5	98,70	0,41
Diaminopimelic acid	Y	GCMS	NO VALUE	200	14,451	13458841,50	23,68	9203132,63	23,13	16473505,11	23,97	17257912,00	24,04	-0,55	0,29	0,36	1,05E-01	5,92E-02	2,81E-02	C00666	190,0954	C7H14N2O4	NO VALUE	NO VALUE
4-hydroxyproline	Y	GCMS	NO VALUE	73	13,221	1727048,25	20,72	1590044,43	20,60	1745976,86	20,74	2298771,88	21,13	-0,12	0,02	0,41	7,01E-01	3,97E-01	8,30E-02	C01015	131,0582	C5H9NO3	NO VALUE	NO VALUE
Acetol	Y	GCMS	NO VALUE	73	16,002	1525788,63	20,54	951155,88	19,86	900559,67	19,78	1222976,88	20,22	-0,68	-0,76	-0,32	7,44E-03	1,66E-03	1,22E-01	C05235	74,0368	C3H6O2	NO VALUE	NO VALUE
Adenosine	Y	GCMS	NO VALUE	73	23,768	801610,67	19,61	440911,75	18,75	653838,83	19,32	1239877,43	20,24	-0,86	-0,29	0,63	1,12E-01	4,99E-01	2,15E-01	C00212	267,0967	C10H13N5O4	NO VALUE	NO VALUE
Alanine	Y	GCMS	NO VALUE	116	7,508	37410799,00	25,16	32666366,50	24,96	24694812,14	24,56	40735588,75	25,28	-0,20	-0,60	0,12	7,21E-01	2,89E-02	7,21E-01	C01401	89,0477	C3H7NO2	NO VALUE	NO VALUE
Aminomalonic acid	Y	GCMS	NO VALUE	73	12,546	3403172,00	21,70	1384811,63	20,40	2354421,00	21,17	2040236,50	20,96	-1,30	-0,53	-0,74	3,11E-04	5,92E-02	6,99E-03	C00872	119,0219	C3H5NO4	NO VALUE	NO VALUE
Arachidic acid	Y	GCMS	NO VALUE	369	22,263	591603,25	19,17	571108,75	19,12	389202,67	18,57	614104,43	19,23	-0,05	-0,60	0,05	8,42E-01	1,37E-02	8,17E-01	C06425	312,3028	C20H40O2	NO VALUE	NO VALUE
Cholesterol	Y	GCMS	NO VALUE	129	27,451	812587,63	19,63	482049,43	18,88	781542,00	19,58	1026882,75	19,97	-0,75	-0,06	0,34	3,13E-02	3,21E-01	2,07E-02	C00187	386.354858	C27H46O	NO VALUE	NO VALUE
Cumic acid	Y	GCMS	NO VALUE	73	5,928	2689798,33	21,36	3385291,50	21,69	3504473,86	21,74	4769472,88	22,19	0,33	0,38	0,83	4,78E-01	4,33E-01	1,04E-01	CHEBI:23412	164,0837	C10H12O2	NO VALUE	NO VALUE
Ergosterol	Y	GCMS	NO VALUE	363	28,402	34400308,25	25,04	28471252,88	24,76	29643197,11	24,82	36668373,50	25,13	-0,27	-0,21	0,09	9,59E-01	4,64E-02	5,53E-01	C01694	396.339203	C28H44O	NO VALUE	NO VALUE
Glycerol 1-phosphate	Y	GCMS	NO VALUE	73	15,954	6013306,13	22,52	4666824,75	22,15	4038833,11	21,95	6296907,38	22,59	-0,37	-0,57	0,07	2,30E-01	4,25E-03	7,78E-01	C00623 C03189	172,0137	C3H9O6P	NO VALUE	NO VALUE
Glycine	Y	GCMS	NO VALUE	174	10,352	9360758,13	23,16	6262700,88	22,58	7237126,33	22,79	9684969,13	23,21	-0,58	-0,37	0,05	5,02E-02	4,29E-02	4,66E-02	C00037	75.032028	C2H5NO2	NO VALUE	NO VALUE
Hypoxanthine	Y	GCMS	NO VALUE	73	16,410	2174589,29	21,05	2973598,00	21,50	1781844,29	20,76	4283771,00	22,03	0,45	-0,29	0,98	3,24E-01	4,94E-01	1,23E-02	C00262	136,0385	C5H4N4O	NO VALUE	NO VALUE
Leucine	Y	GCMS	NO VALUE	158	9,865	7182119,75	22,78	3783504,13	21,85	4870283,00	22,22	7213160,50	22,78	-0,92	-0,56	0,01	6,99E-03	7,70E-02	9,85E-01	C16439	131,0946	C6H13NO2	NO VALUE	NO VALUE
Linoleic acid	Y	GCMS	NO VALUE	75	20,320	26396778,25	24,65	16695489,50	23,99	20368504,89	24,28	30063138,75	24,84	-0,66	-0,37	0,19	9,42E-03	1,06E-01	6,45E-01	C01595	280.240234	C18H32O2	NO VALUE	NO VALUE
Methyl linolenate	Y	GCMS	NO VALUE	75	20,376	9026657,50	23,11	4970477,13	22,24	5730298,44	22,45	3843362,71	21,87	-0,86	-0,66	-1,23	8,21E-03	1,20E-02	1,30E-03	HMDB34154	292,2402	C19H32O2	NO VALUE	NO VALUE
Methyl Stearate	Y	GCMS	NO VALUE	74	19,580	4852977,38	22,21	4557222,88	22,12	4881751,22	22,22	5147892,25	22,30	-0,09	0,01	0,09	2,06E-01	9,40E-01	3,75E-01	C00137	298,2872	C19H38O2	NO VALUE	NO VALUE
Myo-inositol	Y	GCMS	NO VALUE	73	19,254	31697875,25	24,92	22828235,38	24,44	25941593,22	24,63	32868075,25	24,97	-0,47	-0,29	0,05	3,16E-02	3,60E-02	5,05E-01	C06424	180,0634	C6H12O6	NO VALUE	NO VALUE
Myristic acid	Y	GCMS	NO VALUE	73	16,851	1557395,00	20,57	1077583,86	20,04	1106582,67	20,08	1548240,75	20,56	-0,53	-0,49	-0,01	5,10E-02	3,44E-04	9,63E-01	544-63-8	228.208923	C14H28O2	NO VALUE	NO VALUE
Oleic acid	Y	GCMS	NO VALUE	73	20,424	1696598,38	20,69	966133,14	19,88	519036,13	18,99	858084,13	19,71	-0,81	-1,71	-0,98	2,32E-01	2,95E-03	7,21E-01	C00712	282,2559	C18H34O2	NO VALUE	NO VALUE
O-phosphocolamine	Y	GCMS	NO VALUE	73	16,186	375440,00	18,52	376888,00	18,52	128028,75	16,97	361826,14	18,46	0,01	-1,55	-0,05	9,76E-01	3,49E-04	7,82E-01	C00346	141,0191	C2H8NO4P	NO VALUE	NO VALUE
Palmitic acid	Y	GCMS	NO VALUE	73	18,796	3339439,00	21,67	2025262,13	20,95	2592005,89	21,31	3403984,63	21,70	-0,72	-0,37	0,03	1,19E-02	1,24E-02	8,80E-01	C00249	256.240234	C16H32O2	NO VALUE	NO VALUE
Phenylethalonamine	Y	GCMS	NO VALUE	174	9,815	8279491,88	22,98	7212301,00	22,78	8893704,22	23,08	10369286,00	23,31	-0,20	0,10	0,32	2,37E-01	2,41E-01	1,48E-02	C02735	137,0841	C8H11NO	NO VALUE	NO VALUE
Phosphate	Y	GCMS	NO VALUE	299	9,887	221084840,00	27,72	143744933,00	27,10	177302020,44	27,40	206220380,00	27,62	-0,62	-0,32	-0,10	6,24E-03	4,59E-03	4,92E-01	C00009	97,9769	H3O4P	NO VALUE	NO VALUE
Proline	Y	GCMS	NO VALUE	156	13,022	972326,50	19,89	477572,25	18,87	11149888,88	23,41	36252337,00	25,11	-1,03	3,52	5,22	1,04E-02	7,98E-01	1,42E-01	30274-77-2	115.063332	C5H9NO2	NO VALUE	NO VALUE
Ribose	Y	GCMS	NO VALUE	73	15,042	1190008,00	20,18	701559,71	19,42	865350,75	19,72	1247162,88	20,25	-0,76	-0,46	0,07	1,10E-02	5,36E-01	8,03E-01	C08353	150,0528	C5H10O5	NO VALUE	NO VALUE
Stearic acid	Y	GCMS	NO VALUE	73	20,597	18303467,50	24,13	12284465,13	23,55	11935237,78	23,51	17622481,50	24,07	-0,58	-0,62	-0,05	4,35E-02	9,64E-04	7,54E-01	C01530	284,2715	C18H36O2	NO VALUE	NO VALUE
Succinic acid	Y	GCMS	NO VALUE	147	10,446	7447558,75	22,83	8004095,25	22,93	8735804,44	23,06	10455555,00	23,32	0,10	0,23	0,49	6,97E-01	1,71E-01	1,88E-02	C00042	118,0266	C4H6O4	NO VALUE	NO VALUE
Uracil	Y	GCMS	NO VALUE	241	10,764	3196680,88	21,61	2346802,88	21,16	2050337,25	20,97	3022786,00	21,53	-0,45	-0,64	-0,08	2,34E-01	1,55E-04	3,28E-01	C00106	112,0273	C4H4N2O2	NO VALUE	NO VALUE
Valine	Y	GCMS	NO VALUE	144	9,110	23553067,00	24,49	19715482,25	24,23	15811252,38	23,91	22306854,88	24,41	-0,26	-0,57	-0,08	9,59E-01	2,26E-03	1,30E-01	C16436	117,0790	C5H11NO2	NO VALUE	NO VALUE
18-carboxy dinor Leukotriene B4	Putative	HPLC (-)	338.1701@4.173488	338,1701	4,17	24729,33	14,59	28133,60	14,78	25226,00	14,62	25571,50	14,64	0,19	0,03	0,05	4,35E-02	6,46E-01	4,65E-01	CHEBI:63980	338,1729	C18H26O6	56,00	-8,28
8-Oxo-dGTP	Putative	HPLC (-)	522.9895@0.35102043	522,9895	0,35	105800,20	16,69	88797,70	16,44	109612,10	16,74	83669,44	16,35	-0,25	0,05	-0,34	1,40E-01	5,21E-01	2,20E-02	C19967	522,9907	C10H16N5O14P3	73,00	-2,34
Acetostearin	Putative	HPLC (-)	434.325@0.22897954	434,3250	0,23	136236,00	17,06	144635,10	17,14	161077,90	17,30	160849,10	17,30	0,09	0,24	0,24	8,29E-01	8,84E-01	1,07E-02	88234,0000	434,3243	C23H46O7	91,00	1,52
Isooctadecanoic acid	Putative	HPLC (-)	284.2713@8.077122	284,2713	8,08	179692,40	17,46	181578,00	17,47	67153,44	16,04	180116,20	17,46	0,02	-1,42	0,00	7,60E-01	1,01E-12	9,61E-01	C20356	284,2715	C18H36O2	99,00	-0,70
Lyso-Phosphatidylethanolamine	Putative	HPLC (-)	503.3005@5.029128	503,3005	5,03	561455,38	19,10	690366,29	19,40	766893,86	19,55	627474,57	19,26	0,30	0,45	0,16	4,01E-02	4,79E-03	1,52E-01	C00681	503,3012	C25H46NO7P	90,00	-1,35
N-Undecylbenzenesulfonic acid	Putative	HPLC (-)	312.1759@6.93668	312,1759	6,94	145746742,40	27,12	146810750,40	27,13	148587417,60	27,15	146905449,60	27,13	0,01	0,03	0,01	6,23E-01	3,76E-02	6,44E-01	HMDB32549	312,1759	C17H28O3S	97,00	0,00
Phosphatidylinositol	Putative	HPLC (-)	598.3118@6.8272004	598,3118	6,83	34485445,00	25,04	37532470,80	25,16	38259561,00	25,19	30956934,00	24,88	0,12	0,15	-0,16	2,01E-02	3,04E-02	1,06E-02	C05981	598,3118	C27H51O12P	98,00	-0,05
Pseudouridine 5'-phosphate	Putative	HPLC (-)	324.0358@0.27614	324,0358	0,28	264864,20	18,01	242325,30	17,89	255930,10	17,97	198282,20	17,60	-0,13	-0,05	-0,42	9,38E-02	4,12E-01	1,47E-05	C01168	324,0359	C9H13N2O9P	97,00	-0,31
Farnesol	Putative	HPLC (+)	222.1981@6.779746	222,1981	6,779746	154272,13	17,24	157545,44	17,27	122416,40	16,90	154436,11	17,24	0,03	-0,33	0,00	9,44E-01	8,55E-01	1,00E+00	C01126	222,1984	C15H26O	83,00	-1,33
2-Amino-3-methyl-1-butanol	Putative	HPLC (+)	103.0993@5.3992414	103,0993	5,3992414	3904907,44	21,90	4051313,50	21,95	4516304,50	22,11	3987342,80	21,93	0,05	0,21	0,03	9,89E-05	1,57E-03	2,67E-06	CHEBI:28122	103,0997	C5H13NO	46,83	1,78
9(R)-HODE	Putative	HPLC (+)	296.2326@6.5507607	296,2326	6,5507607	427189,13	18,70	548541,60	19,07	552094,89	19,07	479358,11	18,87	0,36	0,37	0,17	5,78E-01	8,55E-01	9,52E-01	LMFA02000036	296,2351	C18H32O3	88,00	-8,57
Glycerophospho-N-Oleoyl Ethanolamine	Putative	HPLC (+)	479.3002@5.779559	479,3002	5,779559	3163819,11	21,59	3007759,00	21,52	3277493,40	21,64	2106328,50	21,01	-0,07	0,05	-0,59	1,09E-02	1,22E-02	7,58E-01	45335	479,3012	C23H46NO7P	93,00	-2,04
Glycidyl oleate	Putative	HPLC (+)	338.2814@7.0782633	338,2814	7,0782633	604290,13	19,20	625380,57	19,25	745199,78	19,51	489281,43	18,90	0,05	0,30	-0,30	9,58E-01	6,93E-01	4,89E-01	C19426	338,2821	Glycidyl oleate	98,00	-2,08
Hexadecasphinganine	Putative	HPLC (+)	273.2663@4.0876594	273,2663	4,0876594	47422,33	15,53	31795,40	14,96	38994,80	15,25	44343,30	15,44	-0,58	-0,28	-0,10	7,11E-01	9,17E-01	1,00E+00	C13915	273,2668	C16H35NO2	66,00	-1,89
N-stearoyl valine	Putative	HPLC (+)	383.3391@7.4144588	383,3391	7,4144588	6399844,56	22,61	6581579,80	22,65	6706293,30	22,68	6544801,40	22,64	0,04	0,07	0,03	7,88E-01	8,49E-01	9,92E-01	75504	383,3399	C23H45NO3	96,00	-2,18
Phosphatidylserine	Putative	HPLC (+)	736.4941@6.5272985	736,4941	6,5272985	55383,25	15,76	37731,00	15,20	49798,90	15,60	57623,44	15,81	-0,55	-0,15	0,06	3,16E-01	9,17E-01	1,00E+00	C02737	736,5003	C37H73N2O10P	70,00	-8,43
Sebacic acid	Putative	HPLC (-)	202.1201@3.0770624	202,1201	3,08	62545,60	15,93	62823,00	15,94	56476,33	15,79	61840,70	15,92	0,01	-0,15	-0,02	9,27E-01	4,35E-02	7,95E-01	C08277	202,1205	C10H18O4	83,00	-1,98
